# Rapid adaptation of predictive models during language comprehension: Aperiodic EEG slope, individual alpha frequency and idea density modulate individual differences in real-time model updating

**DOI:** 10.3389/fpsyg.2022.817516

**Published:** 2022-08-26

**Authors:** Ina Bornkessel-Schlesewsky, Isabella Sharrad, Caitlin A. Howlett, Phillip M. Alday, Andrew W. Corcoran, Valeria Bellan, Erica Wilkinson, Reinhold Kliegl, Richard L. Lewis, Steven L. Small, Matthias Schlesewsky

**Affiliations:** ^1^Cognitive Neuroscience Laboratory, Australian Research Centre for Interactive and Virtual Environments, University of South Australia, Adelaide, SA, Australia; ^2^Innovation, Implementation and Clinical Translation (IIMPACT) in Health, University of South Australia, Adelaide, SA, Australia; ^3^Beacon Biosignals, Boston, MA, United States; ^4^Cognition and Philosophy Laboratory, Monash University, Melbourne, VIC, Australia; ^5^Monash Centre for Consciousness and Contemplative Studies, Monash University, Melbourne, VIC, Australia; ^6^Division of Training and Movement Science, University of Potsdam, Potsdam, Germany; ^7^Department of Psychology, University of Michigan, Ann Arbor, MI, United States; ^8^Weinberg Institute for Cognitive Science, University of Michigan, Ann Arbor, MI, United States; ^9^School of Behavioral and Brain Sciences, University of Texas at Dallas, Dallas, TX, United States

**Keywords:** language comprehension, predictive coding, precision, EEG, N400, aperiodic slope, idea density, individual alpha frequency (IAF)

## Abstract

Predictive coding provides a compelling, unified theory of neural information processing, including for language. However, there is insufficient understanding of how predictive models adapt to changing contextual and environmental demands and the extent to which such adaptive processes differ between individuals. Here, we used electroencephalography (EEG) to track prediction error responses during a naturalistic language processing paradigm. In Experiment 1, 45 native speakers of English listened to a series of short passages. Via a speaker manipulation, we introduced changing intra-experimental adjective order probabilities for two-adjective noun phrases embedded within the passages and investigated whether prediction error responses adapt to reflect these intra-experimental predictive contingencies. To this end, we calculated a novel measure of speaker-based, intra-experimental surprisal (“speaker-based surprisal”) as defined on a trial-by-trial basis and by clustering together adjectives with a similar meaning. N400 amplitude at the position of the critical second adjective was used as an outcome measure of prediction error. Results showed that N400 responses attuned to speaker-based surprisal over the course of the experiment, thus indicating that listeners rapidly adapt their predictive models to reflect local environmental contingencies (here: the probability of one type of adjective following another when uttered by a particular speaker). Strikingly, this occurs in spite of the wealth of prior linguistic experience that participants bring to the laboratory. Model adaptation effects were strongest for participants with a steep aperiodic (1/f) slope in resting EEG and low individual alpha frequency (IAF), with idea density (ID) showing a more complex pattern. These results were replicated in a separate sample of 40 participants in Experiment 2, which employed a highly similar design to Experiment 1. Overall, our results suggest that individuals with a steep aperiodic slope adapt their predictive models most strongly to context-specific probabilistic information. Steep aperiodic slope is thought to reflect low neural noise, which in turn may be associated with higher neural gain control and better cognitive control. Individuals with a steep aperiodic slope may thus be able to more effectively and dynamically reconfigure their prediction-related neural networks to meet current task demands. We conclude that predictive mechanisms in language are highly malleable and dynamic, reflecting both the affordances of the present environment as well as intrinsic information processing capabilities of the individual.

## 1. Introduction

Predictive coding (e.g., Friston, 2005, 2009) provides a compelling theory of how the human brain processes information. Within a unified account of sensation, cognition and action (e.g., Clark, 2013), it posits that the brain utilizes generative predictive models to actively infer the causes of its sensory inputs. In other words, perception involves the brain using its internal model of the world to generate predictions about expected upcoming sensory input, which are then compared to the actual incoming sensory signals. In line with the “Bayesian brain hypothesis” (e.g., Knill and Pouget, 2004; Frith, 2007; Sanborn and Chater, 2016), this is viewed as a process of (unconscious) probabilistic inference: the prior belief arising from a probabilistic generative model is combined with the sensory evidence to yield a posterior belief (the updated model). Predictions flow from higher to lower levels of a hierarchically organized cortical architecture (via feedback connections) and prediction errors are propagated up the cortical hierarchy (via feedforward connections) to engender model updates at higher levels. While predictions at “lower” levels pertain directly to specific properties of the incoming sensory information, predictions at higher levels are more abstract and can span longer timescales (Hohwy, 2013). In this highly efficient coding scheme, sensory information need only be represented to the extent that it is not predicted (Rao and Ballard, 1999). In other words, prediction errors serve as a proxy for sensory information (Feldman and Friston, 2010; Clark, 2013)^1^. This effectively amounts to signal compression as only the non-predicted parts of the signal need to be transmitted. Overall, the architecture strives to minimize prediction errors.

Crucially, the relative weighting of a prediction error (PE) vis-à-vis the top-down predictive model depends both on the noisiness of the signal (Clark, 2013) and the (un)certainty of the prediction (Feldman and Friston, 2010; Vilares and Kording, 2011). This is known as precision weighting: precision, which is defined as the inverse of variance, reflects the confidence or certainty associated with a belief or a sensory input (Friston, 2009; Feldman and Friston, 2010; Adams et al., 2013). For example, when the sensory evidence conflicts with a prior belief, the degree to which the prior will be shifted toward the sensory evidence in forming the posterior belief depends on the certainty vested in the sensory signal (for a useful illustration, see Figure 1 in Adams et al., 2013). Thus, high-precision (i.e., low uncertainty) prediction errors are associated with higher gain (Friston, 2009) and consequently have a more substantial impact on model updating.

In addition, previous work suggests that the top-down/bottom-up balance changes across the lifespan (Moran et al., 2014) and in non-neurotypical populations (e.g., schizophrenia; see Fletcher and Frith, 2009; Adams et al., 2013). Moran et al. (2014) show that older adults tend to weight model predictions more strongly than younger adults. This means that, when faced with unpredicted sensory input, older adults will attribute higher precision to prior beliefs vis-à-vis the sensory evidence and thereby show a lower rate of learning or model adaptation than younger adults. Moran and colleagues suggest that this protects against the overfitting of internal models to the input, thus resulting in less complex models. For positive symptoms of schizophrenia (hallucinations and delusions), by contrast, Fletcher and Frith (2009) suggest that these “are caused by an abnormality in the brain's inferencing mechanisms, such that new evidence (including sensations) is not properly integrated, leading to false prediction errors" (p.56). Using simulations, Adams et al. (2013) show that this can be understood as resulting from less precise top-down predictions, thus “rendering everything relatively surprising” (p.13), including sensations that should not be e.g., self-generated actions; see Clark (2015) for detailed discussion.

These observations suggest that different weightings of top-down (prior) and bottom-up (sensory evidence) information can be a source of individual differences in sensory processing/perceptual inference, specifically in regard to how individuals from different populations adapt their predictive models to changing environmental contingencies. With the present study, we aimed to examine whether such inter-individual differences can also be observed in young, healthy adults (i.e., within the population most typically examined in cognitive neuroscience experiments). We used language as a test domain in which to examine this hypothesis. As a means of studying model adaptation, we investigated individual differences in the extent to which language-related brain responses (the N400 event-related potential) adapt to context-specific probabilistic information (“surprisal") as determined by the experimental environment. In the following, we will first introduce prediction-related phenomena in language and how these can be couched within the predictive coding framework, before turning to a discussion of potential predictors for individual differences in predictive language processing. Finally, we introduce the present study and our hypotheses.

### 1.1. Prediction and predictive coding in language

Language involves a plethora of predictable information sources across a range of different levels. Here, we focus mostly on the sentence level, as this is the level of interest to the current study. When words are combined into sentences, inter-word dependencies give rise to predictability in various ways. For examples, see the Supplementary materials. Note that we use predictability here rather than prediction to make clear that we are referring to the probabilistic dependencies within the structure of language rather than any putative processing mechanisms; for overviews of probabilistic modeling in psycholinguistics, see, for example, Jurafsky (2003) and Chater and Manning (2006). Experience-based, probabilistic information sources—for example that a determiner (e.g., “the”) will at some point be followed by a noun (e.g., “apple") - can be used as priors within a predictive coding architecture. This type of approach has been implemented in computational models of language processing focusing on surprisal or other information-theoretic notions (e.g., Hale, 2006; Levy, 2008); for a recent review, see Hale (2016). The notion of surprisal, which reflects how unexpected a word is given the context in which it appears, is closely related to that of prediction errors in predictive coding. Given a sequence of words *w*_1_, *w*_2_, …, *w*_*t*_, the surprisal of word *w*_*t*_ is defined as the negative logarithm of the probability of that word's occurrence, given the preceding words *w*_1_, …, *w*_*t*−1_:


$$surprisal\left(w_{t}\right)=-logP\left(w_{t}|w_{1},\ldots ,w_{t-1}\right)$$

Surprisal has been linked to neurophysiological correlates of language processing, particularly the N400 event-related potential (ERP) component (Frank et al., 2015; Kuperberg, 2016). There have also been explicit attempts to link speech and language processing to predictive coding architectures (e.g., Pickering and Garrod, 2007, 2013; Skipper et al., 2007; Poeppel et al., 2008; Rauschecker and Scott, 2009; Bornkessel-Schlesewsky et al., 2015b). In addition, several studies suggest that probabilistic information regarding higher-order language-related information is used to anticipate sensory input (Dikker et al., 2010; Dikker and Pylkkänen, 2011), a finding which is closely in line with the assumptions of the predictive coding framework.

Nevertheless, prediction as a concept has remained controversial in the cognitive neuroscience of language processing, particularly with regard to the N400; see Kuperberg and Jaeger (2016) for arguments in favor, and Van Petten and Luka (2012) for arguments against. One of the arguments most often used against active prediction—i.e., prediction that goes beyond the preactivation of a word through a semantic network (or similar) and specifically the explicit prediction of a single specific word—is that there is little evidence that N400 amplitude reflects the error signal resulting from a failed prediction. Rather, N400 amplitude appears to be attenuated with increasing predictability. According to Van Petten and Luka (2012), “current data suggest only that N400 amplitudes are reduced in the presence of supportive semantic context and provide little hint that amplitudes are increased when a hypothesis/expectation/prediction is disconfirmed. From our starting premise that predictions should generate both benefits and costs (on different occasions), the apparent absence of costs is problematic" (p.180). They view this as evidence that the N400 reflects (passive) preactivation rather than (active) prediction, with prediction manifesting itself in other ERP components, most notably late positivities with a frontal scalp distribution.

We contend, however, that this pattern of results for the N400 is, in fact, fully in line with the assumptions of a predictive coding model. Recall that, in the typical implementation of this type of model, only error signals are transmitted *via* feedforward connections because predictable sensory input is “canceled out” by top-down activity encoding the relevant predictions. Thus, a reduced signal is transmitted when the input is, to some extent, predictable. By contrast, in the absence of any predictability, the complete sensory information associated with an input item, say a word, needs to be conveyed: an entirely unpredicted/unpredictable word is associated with the largest prediction error signal. When prior context leads to a certain degree of predictability (or preactivation), prediction error is reduced. In this way, we see the attenuation of prediction errors for predicted vs. unpredictable input rather than an increased error signal for a prediction violation (again, in comparison to a context without any predictability). The pattern of N400 effects thus exactly mirrors what one would expect to observe under typical implementations of a predictive coding architecture (for detailed discussion, see Bornkessel-Schlesewsky and Schlesewsky, 2019). Indeed, predictive coding neatly accounts for the well-known observation that N400 amplitude decreases for unexpected words that match the expected word in regard to certain features (e.g., semantic category, Federmeier and Kutas, 1999) or that show a certain degree of form overlap with the expected word (e.g., *via* orthographic neighborhood, Laszlo and Federmeier, 2009, 2011). In these cases, some—but not all—aspects of the incoming input are explained away by the generative predictive model, thereby resulting in an error signal that is intermediary between that for a highly predictable item and an unpredictable item that does not share any features with the most expected continuation. This suggests that the N400 is a composite response that combines error signals at different levels; cf. Bornkessel-Schlesewsky and Schlesewsky (2013), Bornkessel-Schlesewsky and Schlesewsky (2019), and Frank and Willems (2017).

Bornkessel-Schlesewsky and Schlesewsky (2019) proposed that, more specifically, the N400 reflects a precision-weighted error signal. This account builds on the extensive literature linking the mismatch negativity (MMN) to prediction error processing in the auditory domain (e.g., Friston, 2005; Garrido et al., 2009; Moran et al., 2014) and, more specifically, to precision-weighted error responses (Todd et al., 2011, 2013, 2014). By varying the temporal stability of rules underlying the structure of sound sequences, Todd and colleagues showed that prediction-error-related MMN effects respond to the perceived salience of events and that this is influenced both by rule stability and by rule primacy (i.e., which rule was learned first). Bornkessel-Schlesewsky and Schlesewsky (2019) argue that the N400 reflects similar processes but for more complex stimuli—hence its longer latency in comparison to the MMN.

The claim that N400 amplitude correlates with a precision-weighted error signal is supported by several observations. Firstly, N400 effects vary across languages depending on the informativity of a particular feature (e.g., animacy) for sentence-level interpretation in that language (Bornkessel-Schlesewsky and Schlesewsky, 2019, 2020). This provides a natural link to precision weighting: recall that precision is defined as the inverse of variance and variance in the form-to-meaning mapping is clearly reduced for features that are highly informative (cf. work in the context of the Competition Model, e.g., Bates et al., 1982, 2001; MacWhinney et al., 1984). Secondly, N400 amplitude shows a further property that is expected in the context of a precision-weighted error signal account, namely a modulation by attention. As described in detail by Feldman and Friston (2010), selective attention increases the precision associated with an upcoming sensory stimulus. This can lead to an amplification of the prediction error signal. At a microcircuit level, prediction error amplification is thought to be implemented *via* an increased gain of error-encoding units (most likely pyramidal cells in higher cortical layers; cf. Bastos et al., 2012). Similarly, though acknowledging the vastly different level of measurement at play here, N400 amplitude for incongruent (unpredictable) vs. congruent (more predictable) words within a sentence is increased when the attentional focus on a word is increased *via* information structural (focus) and prosodic (accent) information (Wang et al., 2011).

### 1.2. Precision-weighting as a source of inter-individual differences in predictive coding and possible predictors for individual differences in language

We have already sketched out above how precision weighting of prediction errors not only serves to dynamically adapt a predictive coding architecture to the estimated uncertainties of prior expectations and sensory stimuli, but also how such an architecture provides a natural locus for inter-individual differences (e.g., in aging or, in a different manner, in schizophrenia) and that these are measurable using the MMN ERP component. On the basis of the claims by Bornkessel-Schlesewsky and Schlesewsky (2019) about the functional similarity of the MMN and N400, we would also hypothesize the presence of such differences in N400 effects during language processing. Moreover, given that precision weighting of priors and sensory information may plausibly differ between individuals, we will examine whether such differences manifest themselves even in a population typically considered to be relatively homogeneous, namely young healthy adults. In the following, we will introduce the three main measures that we used as predictors of individual differences in the current study: Idea Density, Individual Alpha Frequency and Aperiodic (1/f) Activity.

#### 1.2.1. Idea density

Idea Density (ID; also known as Propositional Density or P-Density: Kintsch and Keenan, 1973) measures the number of ideas expressed relative to the total number of words used, as derived from written or oral text samples. Ideas are operationalised as predicates: for example, verbs, adjectives and negations are all counted as ideas. ID is thought to reflect the efficiency of linguistic information encoding (Cheung and Kemper, 1992; Kemper et al., 2001b; Iacono et al., 2009; Engelman et al., 2010; Farias et al., 2012) and longitudinal evidence shows that ID measures collected from young adults predict cognitive performance in older adulthood (Snowdon et al., 1996). As discussed by Kemper et al. (2001b), ID is not correlated with high school English or maths grades nor with level of educational attainment (see also Ferguson et al., 2014; Spencer et al., 2015). Kemper and colleagues suggest that “low P-Density in young adulthood may reflect suboptimal neurocognitive development, which, in turn, may increase susceptibility to age-related decline due to Alzheimer's or other diseases" (Kemper et al., 2001a, p.602). ID is relatively stable across the adult lifespan but declines in older adulthood (for results from a large-scale study involving texts from over 19,000 respondents, see Ferguson et al., 2014).

Given the link between ID and efficiency of linguistic information encoding, we hypothesized that ID may provide a proxy for the quality of an individual's language model—our rationale being that efficient encoding requires high-quality linguistic representations. If this is indeed the case, high-ID individuals will have a higher precision language model than low-ID individuals and may thus weight model-based predictions more strongly than unexpected input information in the case of a prediction error. This could entail that high-ID individuals adapt their predictive language models more slowly to local contextual affordances than low-ID individuals, in a similar manner to the slower model updating by older adults reported by Moran et al. (2014).

#### 1.2.2. Individual alpha frequency

Evidence is accruing that perception and cognition are discrete rather than continuous (VanRullen, 2016). We perceive the world by discretely sampling sensory input. In the brain, sampling corresponds to oscillations: fluctuations between states of high and low neuronal receptivity, which are coordinated between neurons and neural assemblies to optimize communication between them (Buzsáki and Draguhn, 2004; Fries, 2005). Importantly, the speed of oscillatory activity differs between individuals. In particular, the peak frequency of the dominant alpha rhythm of the human EEG (~8–13 Hz) varies between approximately 9 and 11.5 Hz in young adults (Klimesch, 1999). This variation in *individual alpha frequency* (IAF) is a trait-like characteristic (Grandy et al., 2013b), which shows high heritability (Posthuma et al., 2001; Smit et al., 2006) and test-retest reliability (Gasser et al., 1985; Kondacs and Szabó, 1999). IAF variability has ramifications not only for the alpha band, but also for the adjacent theta (~4–7 Hz) and beta (~15–30 Hz) rhythms. Consequently, IAF determines an individual's sensory sampling rate and this has consequences for the resolution with which sensory input is analyzed and represented. Samaha and Postle (2015) recently reported a compelling demonstration of this relation for the visual modality. They presented participants with two visual flashes in rapid succession and manipulated the inter-stimulus interval (ISI) between them. At very short ISIs, the two visual stimuli fuse into a single percept. Crucially, inter-individual variability in the two-flash-fusion-threshold was correlated with IAF; for a related demonstration of IAF being *causally* related to the length of the temporal window within which multimodal stimuli are integrated with one another, see Cecere et al. (2015).

In addition to correlating with the resolution of sensory sampling, IAF is associated with a range of higher cognitive abilities. High-IAF individuals process information more quickly (Surwillo, 1961, 1963), and perform better on memory tasks (Klimesch, 1999) and general intelligence measures (*g*) (Grandy et al., 2013a). For a different result see Ociepka et al. (2022), who found a relationship between IAF and processing speed but not between IAF and general intelligence. IAF decreases with age from young adulthood onwards (Köpruner et al., 1984; Klimesch, 1999), thus accompanying the well-known decline of many cognitive abilities in older adulthood (e.g., Hedden and Gabrieli, 2004; Salthouse, 2011). Previous work also indicates that language processing and language learning strategies differ between high- and low-IAF individuals (Bornkessel et al., 2004; Bornkessel-Schlesewsky et al., 2015a; Kurthen et al., 2020; Nalaye et al., 2022).

On account of its link to the rate of sensory sampling, we hypothesized that IAF may serve as a proxy for the general quality (i.e., resolution, signal-to-noise ratio) of the sensory input, which, in turn, influences more complex aspects of information processing. If true, this would mean that incoming sensory information is associated with a higher precision for high-IAF individuals in comparison to low-IAF individuals. In the case of a prediction error, high-IAF individuals may thus weight unexpected input information more strongly vis-à-vis model predictions than low-IAF individuals. Consequently, high-IAF individuals may adapt their predictive language models more quickly to local contextual affordances than low-IAF individuals.

#### 1.2.3. Aperiodic (1/f) activity

Complementing the examination of individual differences in oscillatory neural activity (e.g., *via* IAF), a growing body of literature has begun to investigate the possible role of individual differences in non-oscillatory (aperiodic) brain activity. Aperiodic activity follows a *P*~1/*f*^β^ power law (He, 2014), where *P* corresponds to power, *f* to frequency and β is the so-called “power-law exponent.” This overall relationship of lower frequencies in the human EEG being associated with higher amplitudes (power) than higher frequencies has long been recognized. Only more recently, however, has it become clear that the power law exponent parameter—which governs the steepness of the power decrease with increasing frequency—changes dynamically depending on a variety of factors including age and task, as well as an individual's cognitive state (e.g., He, 2014; Voytek et al., 2015; Donoghue et al., 2020). In addition to potentially being clinically relevant (He, 2014), this variability may also reveal individual differences in cognitive processing in healthy individuals. For example, Ouyang et al. (2020) reported that, when both aperiodic (1/f) slope and alpha activity were taken into account, aperiodic slope rather than alpha activity predicted individual differences in processing speed for an object recognition task. These authors thus suggest that previous observations of an association between alpha activity and processing speed may have been due to a confound between oscillatory and aperiodic activity in earlier analyses (cf. also Donoghue et al., 2020). In the domain of language processing, Dave et al. (2018) recently observed a modulation of prediction-related N400 effects by 1/f slope such that a steeper slope predicted more pronounced N400 effects. Further, Cross et al. (2022) found that the learning of certain types of grammatical rules in an artificial language is likewise predicted by inter-individual variability in 1/f slope.

Regarding potential mechanisms underlying the effects of aperiodic slope on cognitive processing, one prominent approach posits that steepness of the aperiodic slope reflects the degree of neural noise (Voytek et al., 2015). Specifically, highly synchronous neural spiking (equated with “lower neural noise") is thought to correlate with a steeper 1/f slope, while more asynchronous or aberrant firing (equated with “higher neural noise”) is associated with a flatter slope (Buzsáki et al., 2012; Voytek and Knight, 2015). This notion of neural noise may, in turn, be associated with the balance between excitatory and inhibitory activity within neural networks (e.g., Gao et al., 2017). As Voytek et al. (2015) show, aging is associated with a flattening of the 1/f slope and this physiological change may underlie effects of cognitive aging such as a slowing of processing speed.

It is important to acknowledge that, in the context of aperiodic activity estimates obtained from scalp EEG, any inferences drawn about individual differences in neural noise are indirect and must be viewed with a certain degree of caution. Nevertheless, we believe that the existing literature supports an association between scalp-recorded aperiodic slope estimates and neural noise, albeit indirectly. Freeman and Zhai (2009) successfully simulated 1/f slopes obtained from intracranial EEG *via* a computational model of mutual excitation among pyramidal cells. They concluded that “variation in the observed slope is attributed to variation in the level of the background activity that is homeostatically regulated by the refractory periods of the excitatory neurons” (Freeman and Zhai, 2009, p.97). Voytek et al. (2015) in turn demonstrated that 1/f slope and age show a similar relationship in intracranial and scalp EEG measures, thus supporting the association between scalp-recorded 1/f slope and neural noise.

In the context of the current study, we will examine the proposal by Dave et al. (2018) that more synchronous neural networks—as reflected in a steeper aperiodic slope—are associated with stronger predictive processing. If this proposal holds, we should observe a stronger reliance on top-down predictive models for individuals with a steeper 1/f slope and, consequently, a potentially slower adaptation of internal predictive models to current contextual affordances than for individuals with a shallower 1/f slope.

### 1.3. The present study

The present study examined how ID, IAF and aperiodic activity are related to prediction error signals in language processing. In Experiment 1, participants listened to 150 short passages (approximately 5 sentences in length) while their EEG was recorded. An example passage is given below:

*Example of the passages presented to participants in the current study:*

Florence was enjoying her long-awaited holiday in Singapore with her close friends. One of the activities she was most looking forward to was visiting the zoo, where she had the opportunity to ride a **huge gray elephant**. Although standing in the **warm humid air** was dreadful, being waved to through the enclosure by the zookeeper brought a smile to her face.

The critical passages (60%, i.e., 90 of 150) each contained 2 two-adjective noun phrases (marked in bold in the example above), which could either have an expected (canonical) or unexpected (non-canonical) order (e.g., canonical: “the huge gray elephant”; non-canonical: “the gray huge elephant"; for seminal work on ERP correlates of adjective order variations, see Kemmerer et al., 2007). With this manipulation, we intended to elicit prediction error responses due to the unexpectedness of the non-canonical adjective orders. In addition, we varied the probability of encountering non-canonical adjective orders by means of a speaker manipulation. Specifically, passages were recorded by two male speakers with varying probabilities of canonical orders. Thus, for the “canonical” speaker, approximately 70% of the critical 180 two-adjective noun phrases were presented to participants in canonical order, while for the “non-canonical” speaker, only approximately 30% of adjectives were canonically ordered.

Building on the proposal that N400 amplitude reflects precision-weighted prediction error signals (Bornkessel-Schlesewsky and Schlesewsky, 2019), our primary outcome variable was the amplitude of the N400 event-related potential at the position of the critical second adjective within the two-adjective noun phrases embedded in our passages.

Through our experimental design, we aimed to examine inter-individual differences in the processing of prediction errors elicited by the non-canonical adjective orders. We used IAF, ID and aperiodic activity (1/f) as our primary predictors of individual differences as outlined above but also collected an additional battery of cognitive and linguistic tests (see the Methods section for further details). Furthermore, we included the speaker manipulation as an additional manipulation of prediction precision. Here, our rationale was that the high number of non-canonical adjective orders produced by the non-canonical speaker would call for adaptation of participants' existing language model, according to which a non-canonical order of two adjectives should be unexpected (cf. the notion of “active listening” put forward by Friston et al., 2021). Participants who adapt more quickly to the contingencies of the current input— i.e., more readily adapt their established predictive model in the face of prediction errors—should thus be expected to show N400 responses aligned with the experimental environment rather than their global language experience. As described above, we tentatively hypothesized that this readiness to adapt might be more pronounced in high-IAF and low-ID individuals on account of the high precision of the sensory input or low precision of the predictive language model, respectively. Individuals with a steep 1/f slope were expected to show a similar pattern to individuals with a high ID (i.e., slower model adaptation) on account of the link that has been postulated between lower neural noise (associated with a steeper 1/f slope) and stronger predictive processes (Dave et al., 2018). In spite of these hypotheses, this was an exploratory study given the complexity of the domain under examination and the fact that this research question has not yet been examined to date—neither in the area of language nor with respect to other cognitive domains.

Given the novelty of the research question, we also report a follow-up experiment with a similar experimental design (Experiment 2), in which we examined whether the results of Experiment 1 could be replicated.

## 2. Experiment 1

### 2.1. Methods

#### 2.1.1. Participants

Forty-five young adults (31 female; mean age: 22.9 years, sd: 3.9, range: 18–33) participated in Experiment 1. Participants were right-handed as assessed by the Edinburgh handedness inventory (Oldfield, 1971), native speakers of English who had not learnt another language prior to starting school. They reported having no diagnosis of neurological or psychiatric conditions, normal hearing and normal or corrected-to-normal vision. The experimental protocol was approved by the University of South Australia's Human Research Ethics Committee (protocol number 36348).

#### 2.1.2. Materials

The critical materials for this experiment were 90 short passages (approximately 5 sentences in length), each of which contained two critical two-adjective noun phrases (NPs; e.g., “a huge gray elephant”). Critical NPs occurred at different positions in each passage so that their occurrence would not be predictable. The order of the prenominal adjectives was manipulated such that, in some cases, they adhered to the expected sequence of “value > size > dimension > various physical properties > color” (Kemmerer et al., 2007, p.240). We will refer to adjective orders adhering to this sequencing as canonical (C) in what follows and to those that do not as non-canonical (N). Passages were recorded by two male speakers of Australian English with the probability of adjectives in the critical NPs occurring in a canonical or a non-canonical order manipulated across speakers. Thus, when listening to the passages, participants were exposed to one speaker (henceforth: the canonical speaker) who produced more canonical than non-canonical orders (C:N ratio of 69%:31%) and another speaker (henceforth: the non-canonical speaker) who produced more non-canonical orders (C:N ratio of 31%:69%). To counterbalance the assignment of speakers to passages, we constructed two versions of the critical materials. Thus, canonicity of speaker varied both within subjects and within items, but the (non-canonical vs. canonical) speaker assignment was fixed throughout the course of each session. The distribution of canonical and non-canonical orders across speakers, versions and passages is shown in Table 1.

**Table 1:** Counterbalancing of canonical and non-canonical adjective orders across versions.

**Version**	**Speaker**	**Passages**	**Orders within passage**
1	Canonical	1–45	1–7: NC 8–14: CN 15–21: NN 22–45: CC
1	Non-canonical	46–90	46-52: CN 53–59: NC 60-66: CC 67–90: NN
2	Canonical	46–90	46–52: NC 53–59: CN 60–66: NN 67–90: CC
2	Non-canonical	1–45	1–7: CN 8–14: NC 15–21: CC 22–45: NN

Within each version, the canonical speaker produced 62 canonical and 28 non-canonical adjective orders (CN ratio = 31:69%), while the non-canonical speaker produced 62 non-canonical and 28 canonical adjective orders (CN ratio = 69:31%). Note that passages were presented in a pseudo-randomized order and interspersed with filler passages; i.e., the passage numbers shown here do not reflect the order of presentation. Abbreviations for orders within passages: C = canonical; N = non-canonical, with the first letter referring to the first two-adjective NP within the passage and the second letter referring to the second two-adjective NP.

Each participant listened to the critical passages from one of the two versions interspersed with 60 filler passages in a pseudo-randomized order. The filler passages included a separate experimental manipulation involving passive sentences and relative clauses and did not contain any two-adjective noun phrases. Thus, every participant was presented with 150 passages in total.

To ensure that participants were listening attentively, they were presented with yes-no comprehension questions after approximately 1/3 of passages. An example comprehension question for the passage example above is: “Did the zookeeper wave at Florence?” (correct answer = yes).

#### 2.1.3. Language models

The principal aim of the present study was to examine how individuals differ in the adaptation of their predictive models to the current environment during language processing. To this end, we focused on the processing of the second adjective (ADJ2) in the critical 2-adjective NPs embedded in the passages. We used bigram-based surprisal to quantify predictability of ADJ2 in the context of the preceding adjective. To allow us to estimate predictability at the level of adjective classes, we first established adjective clusters for our materials. This was accomplished using the following procedure, which was implemented in R (R Core Team, 2021) using the tidyverse (Wickham et al., 2019) and tidymodels (Kuhn and Wickham, 2020) collections of packages as well as the packages tidytext (Silge and Robinson, 2016) and widyr (Robinson, 2021). For package version numbers, please see the analysis scripts provided with the raw data (see Data Availability Statement).

**Procedure for determining adjective clusters and calculating cluster-based surprisal:**

**Figure 1:**
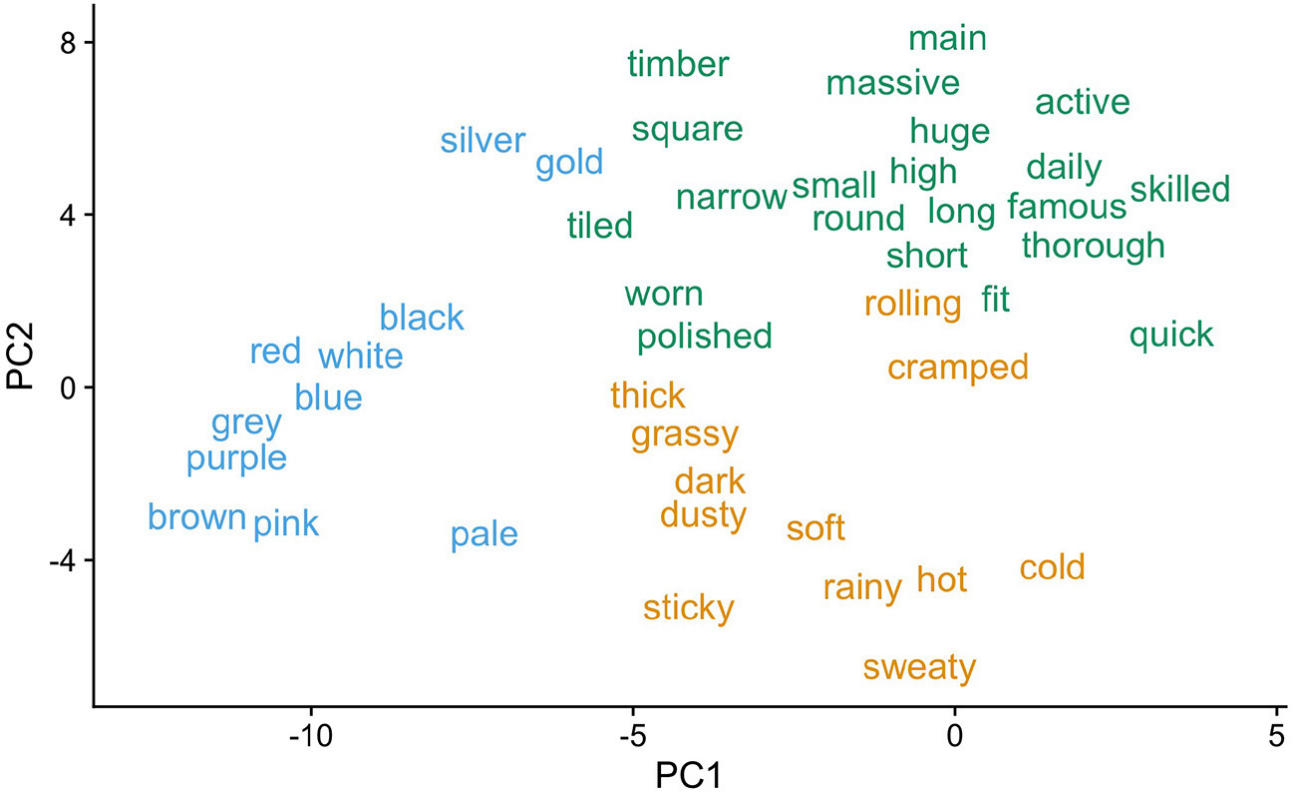
Three adjective clusters produced by the current clustering procedure for Experiment 1. Clusters are visualized with regard to their variability on principal components PC1 and PC2. Note, for example, how the clustering procedure distinguishes color adjectives from other adjective types.

1. We used pre-derived word vectors from van Paridon and Thompson (2021) to determine similarities between adjectives. Word vectors, also known as word embeddings, provide a numerical representation of word meaning. They are created by machine learning models, which learn lexical relationships from word co-occurrences in large text corpora. For a recent example of how word vectors may serve as useful representations of word meaning when investigating human language processing, see Pereira et al. (2018). Here, we used Van Paridon and Thompson's top 1 million vectors from a combined Wikipedia and Open Subtitles corpus.
2. To reduce dimensionality, we performed a principal components analysis (PCA), thus reducing the 300 vectors from van Paridon and Thompson (2021) to 5 principal components (PCs).
3. Six adjective clusters were identified on the basis of the PCs using k-means clustering. The value of k=6 was selected *via* visual inspection of the total within-cluster sum of squares. Three of the six clusters are visualized in Figure 1 and a full list is provided in the Supplementary materials for Experiment 1.
4. Cluster-based unigram and bigram frequencies were computed as cluster-based sums of unigram and bigram counts from the Open Subtitles corpus for English (751 million words) as made available by van Paridon and Thompson (2021). From these, surprisal values for adjective 2 (ADJ2) in the context of adjective 1 (ADJ1) were calculated as:
  $$surprisal(ADJ2)=-log(\frac{ClusterBigramFrequency(ADJ1ADJ2)}{ClusterUnigramFrequency(ADJ1)}$$
5. Here, *ClusterBigramFrequency(ADJ1ADJ2)* refers to the frequency with which two-adjective bigrams comprising a first adjective belonging to the cluster of ADJ1 and a second adjective belonging to the cluster of ADJ2 occur in the Open Subtitles corpus. *ClusterUnigramFrequency(ADJ1)* refers to the frequency with which adjectives belonging to the cluster of ADJ1 occur in Open Subtitle corpus. In the remainder of the paper, we will refer to these corpus-based surprisal values as **global surprisal**.

In a second step, we computed *incremental surprisal* for ADJ2 within the experimental context to be able to track how listeners' expectations change as a function of being exposed to the experimental environment. To track surprisal incrementally over the course of the experiment, we calculated the NP-by-NP cumulative intra-experimental frequencies for the ADJ1-ADJ2 bigram cluster and the ADJ1 unigram cluster and then computed surprisal as described above. This was done separately for each speaker, thus allowing us to examine to what extent participants' expectations adapted to the distributional properties of each of the two speakers within the experiment. We henceforth refer to this speaker-based measure of intra-experimental surprisal as **speaker-based surprisal**. Using speaker-based surprisal, we aimed to examine how participants' N400 responses—as an assumed proxy for precision-weighted prediction error signals— were modulated by the exposure to adjective order variations throughout the course of the experiment and by each speaker.

Corpus-based word (unigram) frequencies for ADJ2 were included in all analyzes as a control variable. These were taken from the same unigram corpus as used for global surprisal calculation above and log-transformed prior to inclusion in the analysis.

#### 2.1.4. Behavioral individual differences measures

##### 2.1.4.1. Idea density (ID)

Participants provided a written text sample in response to the prompt “Describe your favorite game.” This corresponds to the Essay Composition task of the Wechsler Individual Achievement Test—Australian and New Zealand Standardized, Third Edition (WIAT-III A&NZ; Pearson Clinical). From this text, we calculated ID using the automated Computerized Propositional Idea Density Rater (CPIDR; Brown et al., 2008).

##### 2.1.4.2. Cognitive tests

Participants completed an additional battery of cognitive tests. These included:

- The two-subtest version of the Wechsler Abbreviated Scale of Intelligence—Second Edition (WASI-II; Pearson Clinical), comprising Vocabulary and Matrix reasoning tasks
- Three additional language-related subtests from the WIAT-III, namely Oral Word Fluency, Sentence Repetition and Sentence Composition
- A reading-span task (Daneman and Carpenter, 1980)

In accordance with our hypotheses, we focus on ID and the resting state EEG-based individual differences metrics (1/f slope and Individual Alpha Frequency; see below) as our primary measures of individual differences for the purposes of the present study.

#### 2.1.5. Procedure

Participants completed two in-lab testing sessions: (1) a behavioral session comprising the cognitive tests/text sample production, and (2) an EEG session comprising the collection of resting-state EEG recordings as well as the main language comprehension task. Sessions were either completed on the same day, separated by a break (approximately 30 min), or on 2 days (with the second session completed within 7 days of the first session).

##### 2.1.5.1. Behavioral session

In the behavioral session, after the consent process, participants completed a questionnaire to provide demographic, language and well-being details. They subsequently completed the cognitive tests as described above. The behavioral session took maximally 1.5 h to complete.

##### 2.1.5.2. EEG session

In the EEG session, participants were fitted with an EEG cap and underwent a 2-min eyes-open and 2-min eyes-closed resting state EEG recording prior to commencing the main task. For the main task, each trial commenced with the 500 ms presentation of a fixation asterisk in the center of a computer screen, after which the auditory presentation of a passage commenced *via* loudspeakers. After the auditory passage was complete, the fixation asterisk remained on screen for another 500 ms. Subsequently, participants were presented with a comprehension question in approximately 1/3 of all trials, to which they responded with “yes" or “no" using two buttons on a game controller. The assignment of “yes” and “no” responses to the left and right controller buttons was counterbalanced across participants and the maximal response time was set at 4,000 ms. For trials without a comprehension question, participants were asked to “Press the YES key to proceed.” Following the participant's response or after the allocated response time had elapsed, the next trial commenced after an inter-trial interval of 1,500 ms. Participants were asked to avoid any movements or blinks during the presentation of the fixation asterisk if possible.

Note that, as the intermittent comprehension questions only served to ensure that participants listened attentively, comprehension data was not analyzed in the present paper. Log files for the comprehension task are, however, provided with the raw data for the experiment (see Data Availability statement).

The 150 passages were presented in 5 blocks, between which participants took short self-paced breaks. Prior to commencing the main task, participants completed a short practice session. After the main task, the resting state recordings were repeated. Overall, the EEG session took approximately 3 h including electrode preparation and participant clean-up.

#### 2.1.6. EEG recording and preprocessing

The EEG was recorded from 64 electrodes mounted inside an elastic cap (Quik-CapEEG) using a Neuroscan Synamps2 amplifier (Compumedics Neuroscan, Abbotsford, VIC, Australia). The electrooculogram (EOG) was recorded via electrodes placed at the outer canthi of both eyes as well as above and below the left eye. The EEG recording was sampled at 1,000 Hz and referenced to the right mastoid.

Data preprocessing was undertaken using MNE Python version 0.23.0 (Gramfort et al., 2013, 2014). EEG data were re-referenced to an average reference and downsampled to 500 Hz prior to further processing. EOG-artifacts were corrected using an ICA-based correction procedure, with independent components (ICs) found to correlate most strongly with EOG events (via the create_eog_epochs function in MNE) excluded. Raw data were filtered using a 0.1—30 Hz bandpass filter to exclude slow signal drifts and high frequency noise. Epochs were extracted in a time window from –200 to 1,000 ms relative to critical word (ADJ2) onset and mean single-trial amplitudes were extracted for the prestimulus (–200 to 0 ms) and N400 (300–500 ms) time windows using the retrieve function from the philistine Python package (Alday, 2018).

##### 2.1.6.1. Resting-state EEG-based individual differences measures: Individual alpha frequency (IAF) and aperiodic (1/f) activity

IAF and aperiodic slope estimates were calculated from participants' eyes-closed resting-state recordings.

To calculate IAF, we used a Python-based implementation (Alday, 2018) of the procedure described in Corcoran et al. (2018) and drawing on electrodes P1, Pz, P2, PO3, POz, PO4, O1, Oz and O2. We estimated both peak alpha frequency (PAF) and center of gravity (COG) measures (cf. Corcoran et al., 2018, for discussion) and calculated the mean of pre and post estimates by participant for each measure. For participants who did not have estimable IAF values for one of the two recording sessions, their IAF estimate from the other session was used as their overall IAF metric. This was the case for 3 participants in Experiment 1.

Aperiodic (1/f) intercept and slope estimates were calculated in Python using the YASA toolbox (Vallat and Walker, 2021). YASA implements the irregular-resampling auto-spectral analysis (IRASA) method for separating oscillatory and aperiodic activity (Wen and Liu, 2016). As for IAF, by-participant intercept and slope estimates were computed as means of pre and post resting-state recordings from electrodes F7, F5, F3, F1, Fz, F2, F4, F6, F8, FT7, FC5, FC3, FC1, FCz, FC2, FC4, FC6, FT8, T7, C5, C3, C1, Cz, C2, C4, C6, T8, TP7, CP5, CP3, CP1, CPz, CP2, CP4, CP6, TP8, P7, P5, P3, P1, Pz, P2, P4, P6, P8, PO7, PO5, PO3, POz, PO4, PO6, PO8, O1, Oz, and O2.

#### 2.1.7. Data analysis

The data analysis was undertaken using R (R Core Team, 2021) and Julia (Bezanson et al., 2017). We used R for data pre- and post-processing. For data import and manipulation, we used the tidyverse collection of packages (Wickham et al., 2019) as well as the vroom package (Hester and Wickham, 2021). Figures were created using ggplot2 (Wickham, 2016; Wickham et al., 2021) as well as the packages cowplot (Wilke, 2021) and patchwork (Pedersen, 2020). All figures with color employ the Okabe Ito color palette from colorblindr (McWhite and Wilke, 2021). Other packages used include corrr (Kuhn et al., 2020), kableExtra (Zhu, 2021) and here (Müller, 2020). For package version numbers, please see the analysis scripts provided with the raw data (see Data Availability Statement). For R, see the html outputs in the src/subdirectory; for Julia see the Manifest.toml file.

EEG data were analyzed using linear mixed effects models (LMMs) with the MixedModels.jl package in Julia (Bates et al., 2021). We used the JellyMe4 package (Alday, 2021) to move model objects from Julia to R for visualization purposes.

For the ERP data, we examined single-trial N400 amplitude as our outcome variable of interest. To this end, we analyzed mean EEG voltage 300–500 ms post onset of the critical second adjective (ADJ2) in a centro-parietal region of interest (C3, C1, Cz, C2, C4, P3, P1, Pz, P2, P4, CP3, CP1, CPz, CP2, CP4).

##### 2.1.7.1. Linear mixed modeling approach

We adopted a parsimonious LMM selection approach (Bates et al., 2015; Matuschek et al., 2017), which seeks to identify LMMs that are supported by the data and not overparameterized. Model selection was undertaken without consideration of fixed-effects estimates (i.e., without consideration of which fixed effects reached significance).

Fixed effects initially included log-transformed unigram frequency, speaker-based surprisal, adjective order canonicity, epoch (as a proxy for how long participants had been exposed to the experimental stimuli), mean prestimulus amplitude and their interactions. Prestimulus amplitude (–200 to 0 ms) was included as a predictor in the model as an alternative to traditional EEG baselining (see Alday, 2019). The categorical factor canonicity was encoded using sum contrasts (cf. Schad et al., 2020; Brehm and Alday, 2022); thus, model intercepts represent the grand mean. All continuous predictors were z-transformed prior to being included in the models.

Although not of interest within the scope of the current paper, we modeled the main effect of prestimulus amplitude with a second-order and the main effect of speaker-based surprisal with a third-order polynomial trend. The inclusion of these higher-order trends was supported by the data, significantly improved model fit, and guarded against the interpretation of spurious interactions of their linear trends with other fixed effects (Matuschek and Kliegl, 2018). Non-significant higher-order interactions involving fixed effects were removed from the model when they were not part of the theoretical expectations and this did not lead to a significant reduction in goodness of model fit as assessed *via* likelihood-ratio tests (LRTs).

The random-effect (RE) structure was selected in two steps, again using LRTs to check improvement in goodness of fit and random-effects PCA (rePCA) to guard against overparameterization during model selection. The results of the first step led to a RE structure with variance components for grand means, prestimulus amplitude and prestimulus amplitude (2nd order) by subject, item and channel. In a second step, we added by-subject and by-item variance components for effects of canonicity, epoch, unigram frequency and speaker-based surprisal to the RE structure. Correlation parameters were not significant for the by-subject and by-item variance components and constrained to zero.

Using the speaker-based surprisal LMM (as described above) as a reference, we added, in turn, fixed-effect covariates for individual differences in (1) 1/f slope, (2) IAF (peak alpha frequency), and (3) ID to the model to check the extent to which they moderate/modulate adaptation to speaker-based surprisal. In each of these three additional LMMs, adding the respective individual differences covariate as a by-item variance component significantly improved the goodness of model fit.

The model selection procedure is transparently documented in Julia scripts in the Open Science Framework repository for this paper (see Data Availability Statement).

##### 2.1.7.2. Reporting and visualization of results

As our primary research question was how listeners adapt their predictive models to the experimental context, we focus on interactions of speaker-based surprisal and epoch in the interpretation of our results. Thus, for each LMM, we focus on the highest order interaction(s) including these predictors and the current individual-differences predictor of interest where relevant. These are reported, visualized and interpreted in the main text. Model summaries are included in the Supplementary materials, with only significant effects reported in the model summary tables to increase readability. For full model summaries including all effects, see the repository for the paper. For the visualization of effects, we used the broom.mixed package (Bolker and Robinson, 2021) to extract fitted values and the remef package (Hohenstein and Kliegl, 2021) to extract partial effects. By visualizing partial effects, we focus on the effects of interest while adjusting for additional model parameters that are not of primary interest here where appropriate.

### 2.2. Results

#### 2.2.1. Individual differences measures

Distributions of the (z-transformed) individual differences measures are shown in Supplementary Figure S1.

#### 2.2.2. EEG data

##### 2.2.2.1. Sanity check analysis

In a first step, we ran a “sanity check” analysis to determine whether the current data showed expected modulations of N400 amplitude by unigram frequency and global (corpus-based) surprisal defined at the level of adjective clusters (see section on language models above). For this, we followed the general modeling strategy outlined in the Data analysis section above, but including global surprisal rather than speaker-based surprisal.

**Figure 2:**
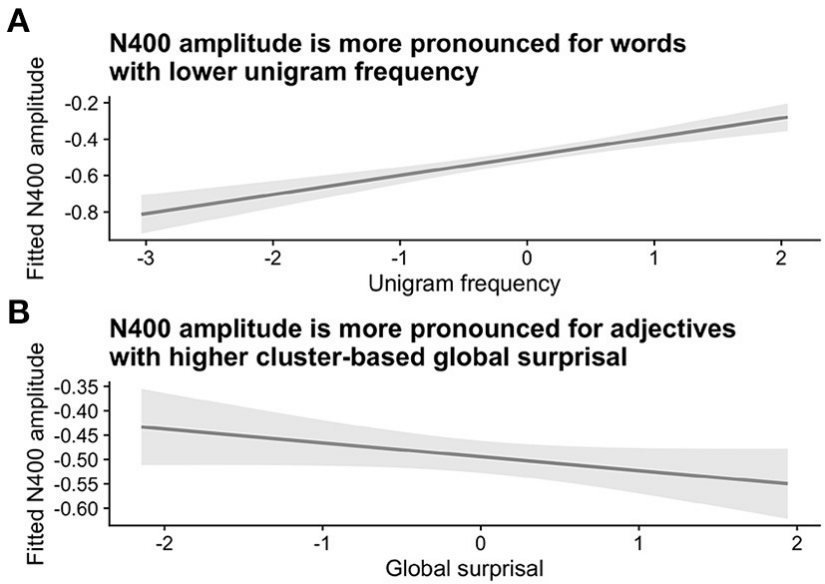
Sanity check analysis for Experiment 1. Panel A shows the relationship between N400 amplitude and (log-transformed) unigram frequency, while Panel B shows the relationship between N400 amplitude and global (corpus-based) surprisal, as defined using bigrams at the adjective cluster level. Both unigram frequency and surprisal values were z-transformed. Shaded areas indicate 95% confidence intervals.

The sanity check analysis confirmed the expected effects of word frequency and surprisal on N400 amplitude. At the position of the critical second adjective, N400 amplitudes were higher for words with a lower frequency of occurrence and for words with higher corpus-based surprisal values. These effects are visualized in Figure 2 (see Supplementary Table S1 for the model summary). As is apparent from the model summary, there was a significant interaction of Unigram Frequency x Global Surprisal x Prestimulus amplitude (Estimate = 0.0497, Std. Error = 0.0203, *z* = 2.45, *p* = 0.01). However, as we were only interested in general trends for word frequency and global surprisal for the purposes of our sanity check, we visualize the partial effects of these two predictors adjusted for the other predictors.

##### 2.2.2.2. N400 amplitude attunes to speaker-based surprisal over the course of the experiment

**Figure 3:**
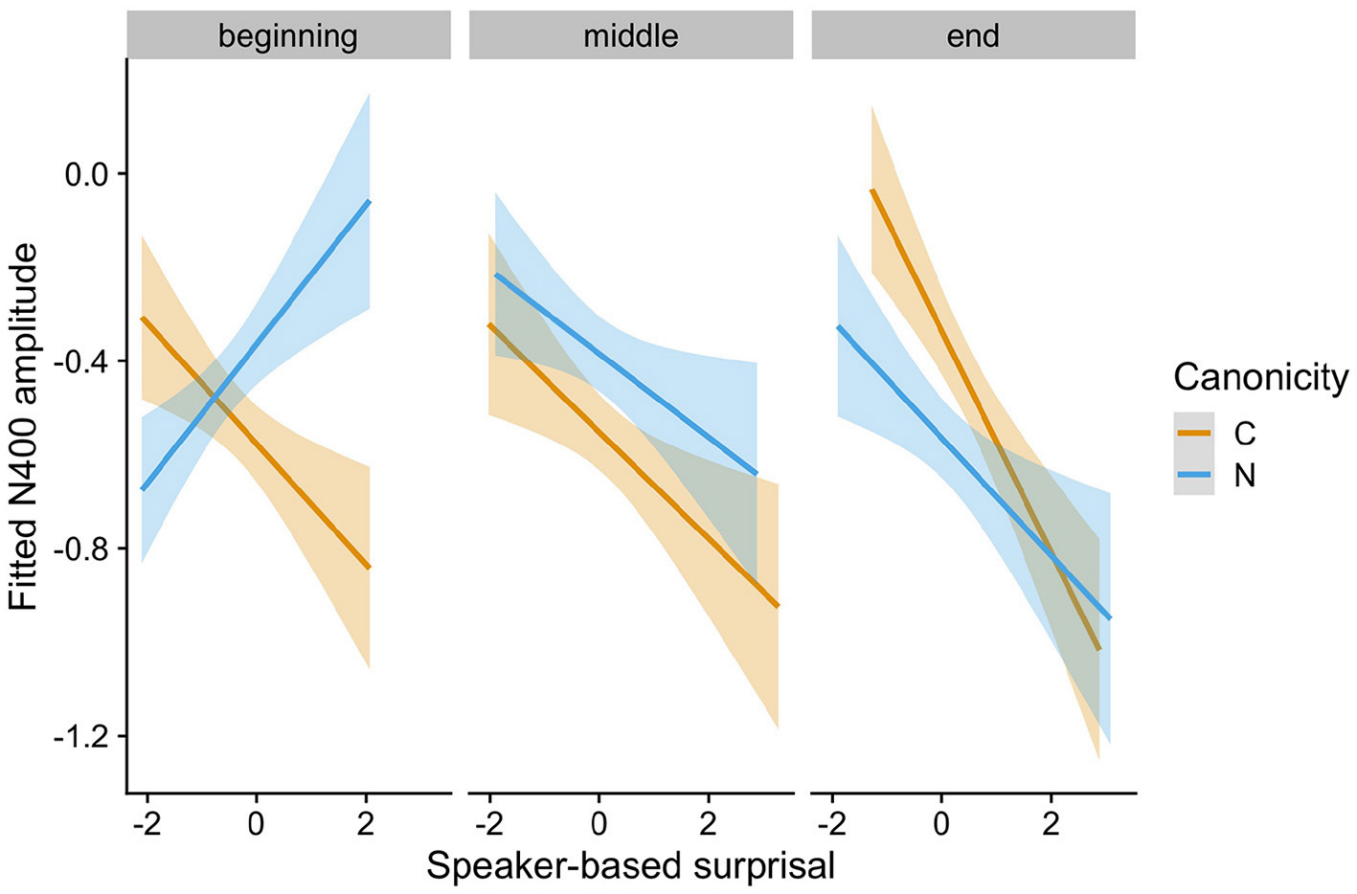
Changes in the relationship between speaker-based surprisal (z-transformed) and N400 amplitude over the course of Experiment 1 for canonical (C) and non-canonical (N) adjective orders. The figure visualizes partial effects as calculated using the *remef* package, adjusted for prestimulus amplitude. Note that position in the experiment (operationalised *via* epoch in the statistical model) is trichotomised into beginning, middle and end for visualization purposes only; epoch was included in the model as a continuous predictor.

The speaker-based surprisal model (see Supplementary Table S2 for the model summary) revealed an interaction of Speaker-based Surprisal x Epoch x Canonicity x Prestimulus Amplitude (Estimate = −0.0693, Std. Error = 0.0173, *z* = −4.01, *p* < 0.0001). Figure 3 visualizes the partial effect of Speaker-based Surprisal x Epoch x Canonicity, adjusted for Prestimulus Amplitude. As is apparent from the figure, the effect of speaker-based surprisal becomes stronger over the course of the experiment, i.e., the longer participants are exposed to the peculiarities of each speaker, the stronger the effect of speaker-based surprisal on N400 amplitude. This supports our assumption that listeners attune their internal predictive models to the current context. Strikingly, the effect of speaker-based surprisal overrides the effect of adjective order canonicity by the end of the experiment [cf. Alday et al. (2017) for the finding that language-related EEG responses adapt to the local context within a story].

##### 2.2.2.3. Inter-individual differences in predictive model adaptation

Having determined that effects of speaker-based surprisal (z-transformed) on N400 amplitude became stronger over the course of the experiment, we next sought to examine how individuals differed with regard to this adaptation process and which of our metrics best predicted these assumed individual differences. To this end, we in turn added each of our individual differences metrics of interest—individual alpha frequency (IAF), aperiodic (1/f) slope and idea density (ID)—to the speaker-based surprisal model without individual differences. As revealed by likelihood ratio tests and goodness-of-fit metrics, all of these models showed an improved fit to the data over the base model without an individual differences predictor. Table 2 provides an overview of the goodness-of-fit metrics, demonstrating that all models including individual differences covariates outperform the model without individual differences in terms of AIC. With the exception of the IAF model, this also holds for BIC.

**Table 2:** Model comparison for the models including speaker-based surprisal in Experiment 1.

	**Model**	**dof**	**Deviance**	**AIC**	**AICc**	**BIC**
1	No Ind. differences	54	513,348	513,456	513,456	513,965
2	Slope	89	512,745	512,923	512,924	513,761
3	IAF	83	513,232	513,398	513,398	514,179
4	ID	84	512,520	512,688	512,688	513,479

The table shows goodness-of-fit metrics for the best-fitting model without individual differences in comparison to the models including aperiodic (1/f) slope, IAF, and ID, respectively. Note that the differing degrees of freedom for the individual-differences models are a result of the pruning of non-significant higher-order interactions from some models; see Data Analysis section for details.

In line with our primary research question, for the interpretation of the individual differences results, we focus on the top-level interaction(s) involving Speaker-based Surprisal, Epoch and the individual differences predictor of interest (cf. the discussion of our LMM modeling approach in the Data Analysis section).

For the model including aperiodic slope, the top-level interaction was Prestimulus Amplitude x Speaker-based Surprisal x Epoch x Canonicity x Frequency x Slope (Estimate = 0.1531, Std. Error = 0.0780, *z* = 1.96, *p* < 0.05). For the model including IAF, it was Speaker-based Surprisal x Epoch x Canonicity x Frequency x IAF (Estimate = –0.0505, Std. Error = 0.0172, *z* = –2.94, *p* < 0.01). The ID model showed an interaction of Prestimulus Amplitude x Speaker-based Surprisal x Epoch x Canonicity x ID (Estimate = 0.0821, Std. Error = 0.0163, *z* = 5.03, *p* < 0.0001). In view of the complexity of these models and the fact that our primary interest for the purposes of the present paper lies in examining how adaptation to speaker-based surprisal is modulated by these individual differences metrics, we visualize partial effects of Speaker-based Surprisal x Epoch x Individual Differences Covariate of Interest for each model in turn in the following, adjusting for any additional moderating effects. For model summaries, see Supplementary Tables S3–S5.

**Figure 4:**
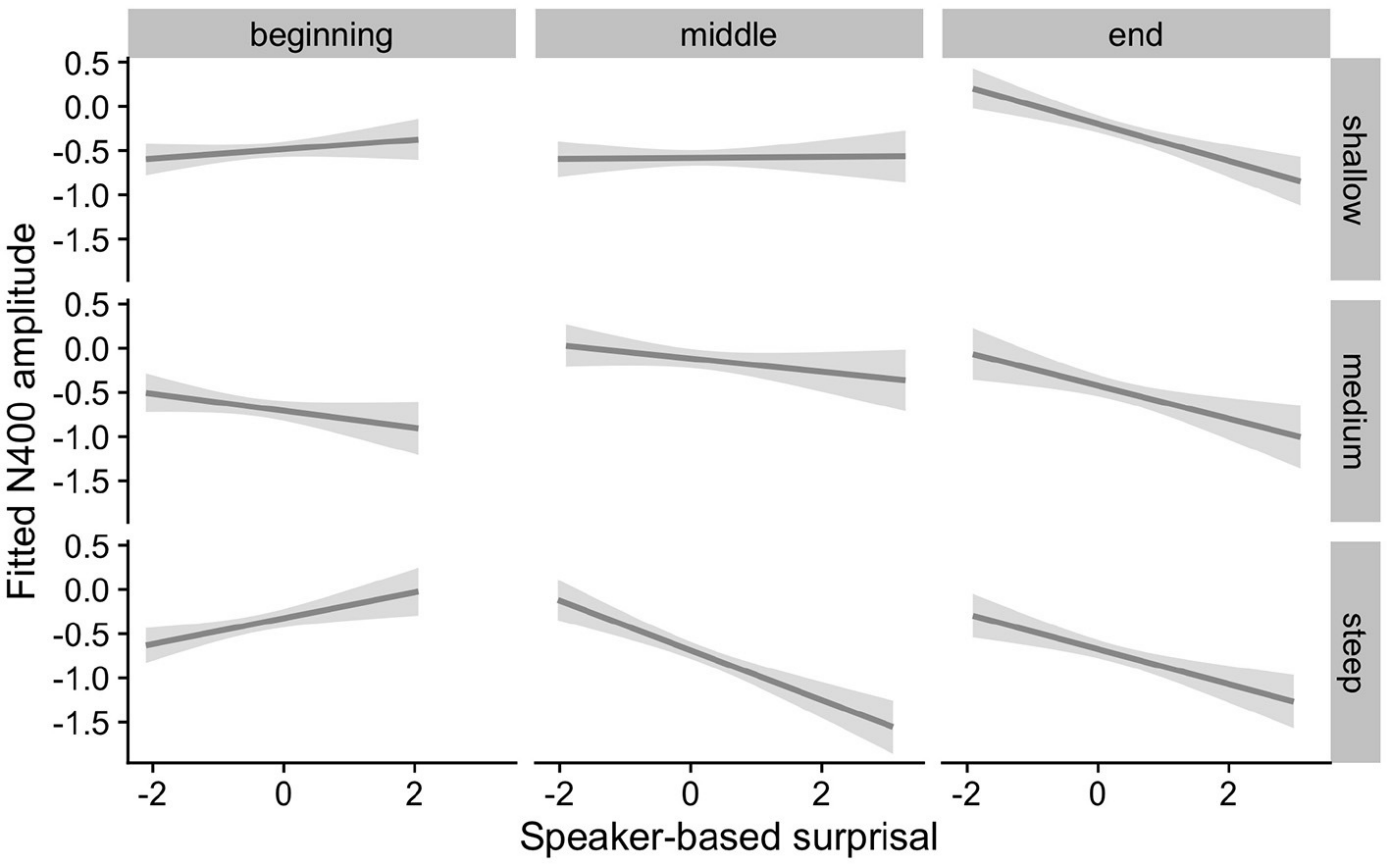
Effect of aperiodic (1/f) slope on changes in the relationship between speaker-based surprisal (z-transformed) and N400 amplitude over the course of Experiment 1. The figure visualizes partial effects as calculated using the *remef* package, adjusted for prestimulus amplitude, canonicity and frequency. Note that position in the experiment (operationalised *via* epoch in the statistical model) is trichotomised (into beginning, middle, end) for visualization purposes only; epoch was included in the model as a continuous predictor. The same holds for 1/f slope, which is trichotomised into steep, medium and shallow for visualization purposes but was entered into the statistical model as a continuous predictor. Shaded areas indicate 95% confidence intervals.

Figure 4 visualizes how the intra-experimental adaptation to speaker-based surprisal is modulated by aperiodic slope. It demonstrates that, though N400 responses had attuned to speaker-based surprisal for all participants by the end of the experiments (mirroring the effects observed in Figure 3), individuals with a steep aperiodic slope adapt most rapidly to intra-experimental contingencies (cf. the pattern of N400 responses in the middle portion of the experiment).

**Figure 5:**
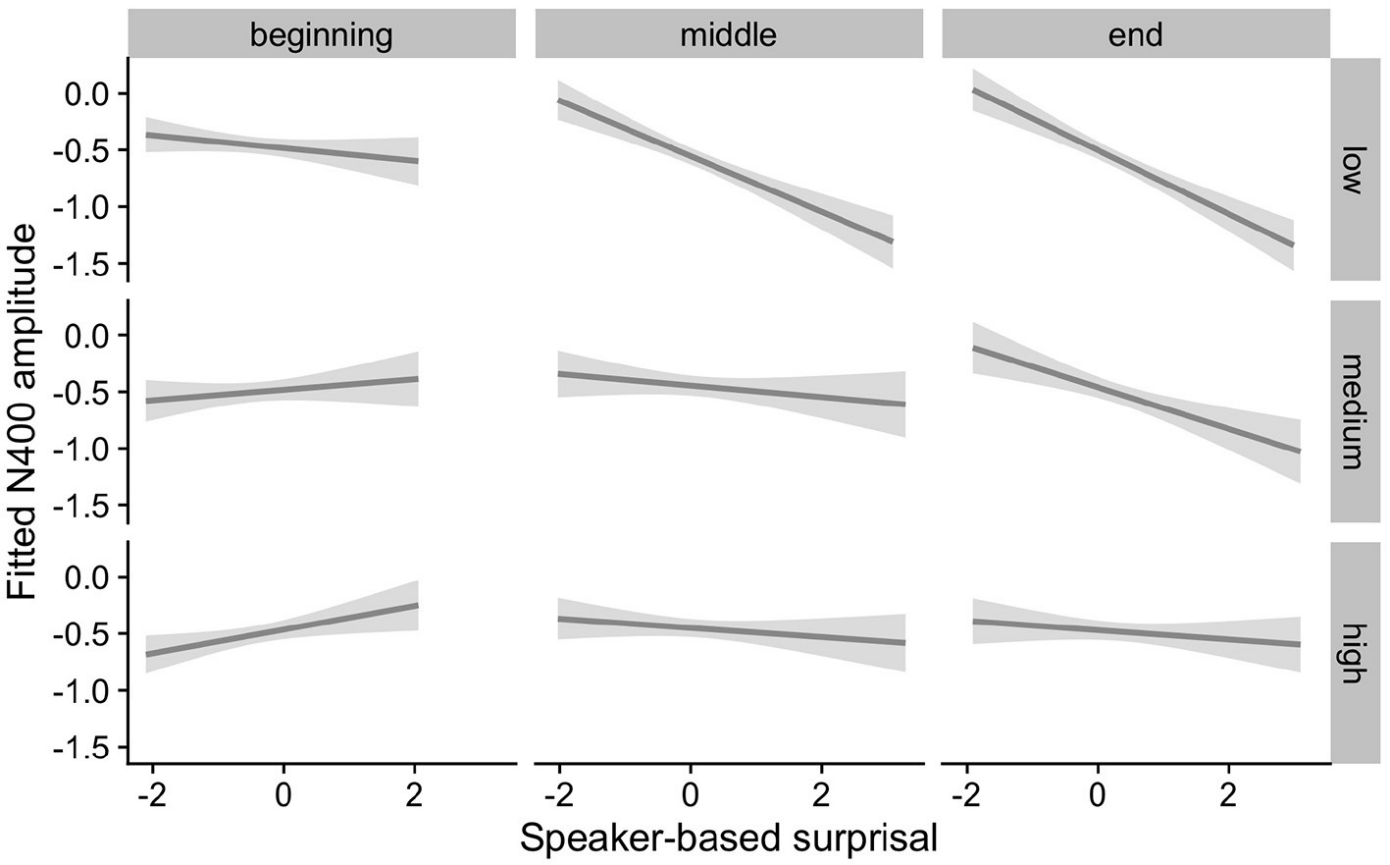
Effect of individual alpha frequency (IAF) on changes in the relationship between speaker-based surprisal (z-transformed) and N400 amplitude over the course of Experiment 1. The figure visualizes partial effects as calculated using the *remef* package, adjusted for canonicity and frequency. Note that position in the experiment (operationalised *via* epoch in the statistical model) is trichotomised into beginning, middle and end for visualization purposes only; epoch was included in the model as a continuous predictor. The same holds for IAF, which is trichotomised into low, medium and high for visualization purposes but was entered into the statistical model as a continuous predictor. Shaded areas indicate 95% confidence intervals.

**Figure 6:**
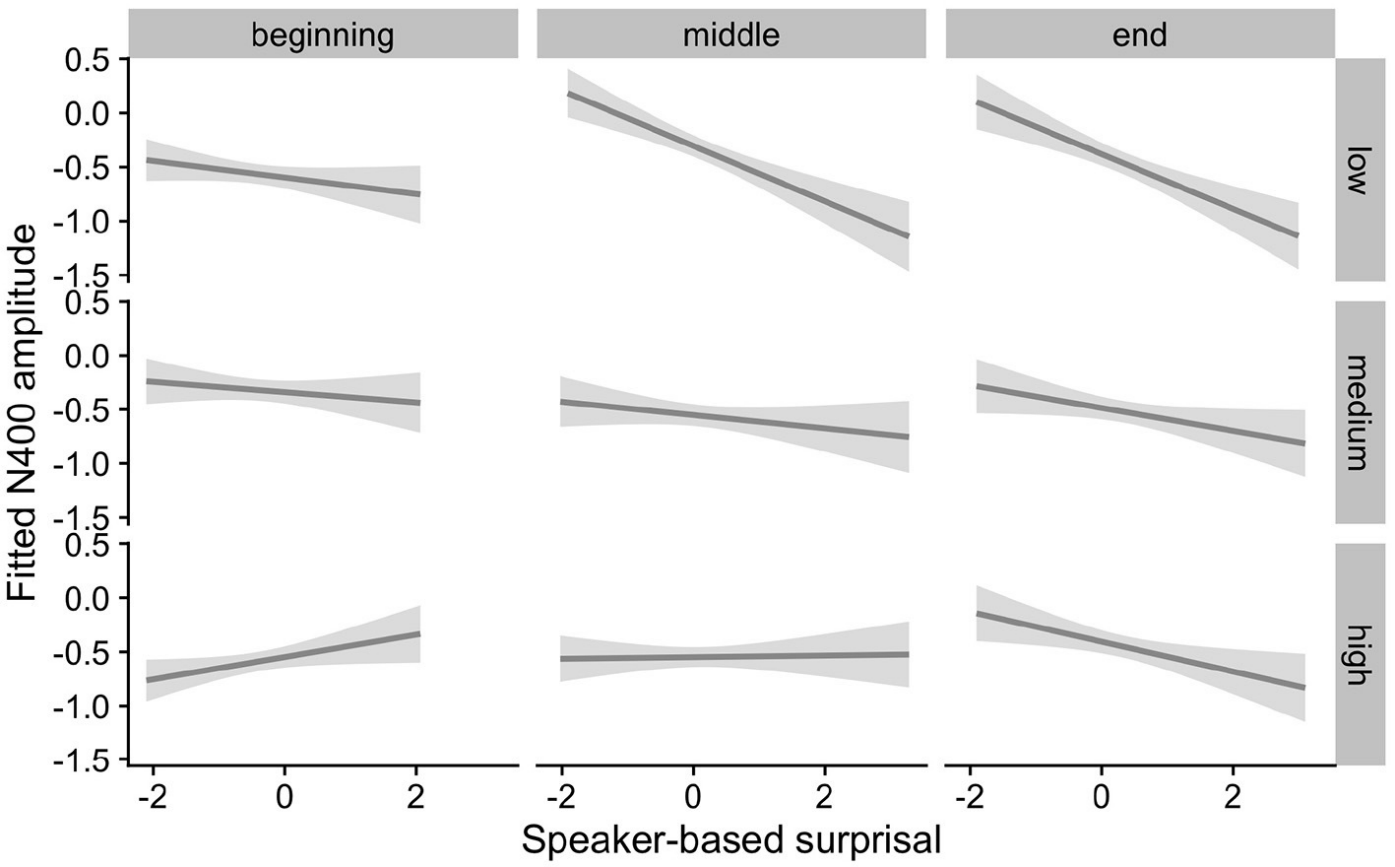
Effect of idea density (ID) on changes in the relationship between speaker-based surprisal (z-transformed) and N400 amplitude over the course of Experiment 1. The figure visualizes partial effects as calculated using the *remef* package, adjusted for prestimulus amplitude and canonicity. Note that position in the experiment (operationalised *via* epoch in the statistical model) is trichotomised into thirds (beginning, middle, end) for visualization purposes only; epoch was included in the model as a continuous predictor. The same holds for ID, which is trichotomised into low, medium and high for visualization purposes but was entered into the statistical model as a continuous predictor. Shaded areas indicate 95% confidence intervals.

Figures 5, 6 show the adaptation to speaker-based surprisal as moderated by IAF and ID, respectively. For IAF, it is apparent that adaptation is quickest for individuals with a low IAF. At a first glance, the pattern is similar for ID, i.e., low-ID individuals show a more rapid adaptation to speaker-based surprisal. However, it is notable that individuals with a high ID show the most pronounced change in the *pattern* of speaker-based surprisal N400 effects over the course of the experiment, demonstrating a slight “anti surprisal" effect at the beginning of the experiment but adapting to show the expected attunement to speaker-based surprisal by the end.

### 2.3. Discussion

Experiment 1 examined N400 ERP responses to investigate how, during naturalistic language processing, individuals update their internal predictive models to reflect current contextual or environmental information. While listening to short passages recorded by two speakers of Australian English, participants showed an adaptation to experiment- and speaker-specific adjective order patterns with increasing exposure to these patterns over the course of the experiment. By the end of the experiment, N400 responses at the position of the critical second adjective (ADJ2) in two-adjective noun phrases embedded in the passages had attuned to speaker-based surprisal. In other words: N400 amplitude reflected the (information-theoretic) surprisal for encountering an adjective of type ADJ2 following an adjective of the type encountered at the ADJ1 position, given the speaker reading the passage. Adjective type was defined using a word-vector-based clustering procedure and speaker-based surprisal was defined incrementally *via* the participant's prior exposure to two-adjective noun phrases for a particular speaker at each point over the course of the experiment. N400 attunement to speaker-based surprisal led to an alignment of N400 amplitudes for canonical and non-canonical adjective orders by the end of the experiment. It is important to keep in mind, however, that these measures (i.e., adjective clusters and surprisal) were correlations rather than experimental manipulations.

In addition, we observed inter-individual differences in regard to the strength of N400-attunement to speaker-based surprisal. All three individual differences predictors examined—aperiodic (1/f) slope, Individual Alpha Frequency (IAF) and Idea Density (ID)—led to improvement of mixed model fit over the best model not including individual differences predictors. Individuals with a steep aperiodic slope, which is thought to reflect low neural noise, showed the most pronounced and earliest attunement to speaker-based surprisal. A similar pattern was observed for individuals with a low IAF. For ID, the pattern was somewhat more mixed: while low-ID individuals appeared to show an earlier attunement to speaker-based surprisal, high-ID individuals showed a more substantial change of speaker-surprisal-related response from the beginning to the end of the experiment. These findings were examined further in Experiment 2.

## 3. Experiment 2

### 3.1. Methods

In view of the exploratory nature of the current study and the novel results of Experiment 1, we ran a second Experiment to determine whether these results could be replicated. Experiment 2 employed a very similar design to Experiment 1 with a new sample of young adults as participants.

#### 3.1.1. Participants

Forty young adults (mean age: 23.8 years, sd: 6.3, range: 18–39) participated in Experiment 2, with 30 identifying as female, 9 identifying as male and 1 identifying as other. Inclusion and exclusion criteria were as for Experiment 1 and the experiment was approved under the same protocol by the University of South Australia's Human Research Ethics Committee. None of the participants for Experiment 2 had taken part in Experiment 1.

#### 3.1.2. Materials

Participants again listened to 150 short passages in Experiment 2, which were adapted from those used in Experiment 1. In contrast to Experiment 1, in which only 90 of the 150 passages contained two critical two-adjective NPs, in Experiment 2, all 150 passages contained two critical NPs. This change was incorporated in order to increase the number of critical items per participant and thus improve our ability to track changes in N400 activity across the course of the experiment. In addition, we made minor modifications to some of the critical NPs from Experiment 1. As for Experiment 1, the full experimental materials are available on the study repository (see Data Availability statement).

The passages were again recorded by two male speakers of Australian English, one of which had already been one of the speakers for Experiment 1. As for Experiment 1, one of the speakers (the “canonical speaker") had a higher probability of producing canonical vs. non-canonical two-adjective orders (approximately 70%:30%), while the other (the “non-canonical speaker") had a lower probability of producing canonical vs. non-canonical orders (approximately 30%:70%). The assignment of speaker to the canonical or non-canonical role was counterbalanced across participants. In order to further accentuate the speaker-specific adjective order characteristics, presentation of the two speakers was alternated in a block-based manner in this experiment. The experiment commenced with one block of the canonical speaker, followed by two blocks of the non-canonical speaker and two further blocks of the canonical speaker.

Comprehension questions were again presented after approximately 1/3 of all passages.

#### 3.1.3. Language models

Adjective clusters and speaker-based surprisal were calculated following the same procedure as for Experiment 1. The adjective clusters for Experiment 2 are listed in the Supplementary materials.

#### 3.1.4. Behavioral individual differences measures

##### 3.1.4.1. Idea density

Participants were given 10 min to produce a written text sample of approximately 300 words in response to the prompt “Describe an unexpected event in your life.” ID was calculated as in Experiment 1.

##### 3.1.4.2. Cognitive tests

Participants completed an additional battery of cognitive tests. These included:

- The four-subtest version of the Wechsler Abbreviated Scale of Intelligence—Second Edition (WASI-II; Pearson Clinical), comprising Block design, Vocabulary, Matrix reasoning and Similarities tasks
- Three subtests from the Test of Adolescent and Adult Language-Fourth Edition (TOAL-4), namely Word opposites, Derivations and Spoken analogies
- Semantic and phonological verbal fluency tasks
- A computer-based hearing test to measure pure-tone hearing thresholds (pure-tone audiometry)

As for Experiment 1, we focus on ID and the resting state EEG-based individual differences metrics (1/f slope and Individual Alpha Frequency, IAF; see below) as our primary measures of individual differences.

#### 3.1.5. Procedure

The two in-lab testing sessions (behavioral and EEG) for Experiment 2 were comparable to those in Experiment 1. The procedure for the EEG testing session was also identical to that for Experiment 1 with two exceptions. Firstly, participants completed a short (approximately 3.5 min) passive auditory oddball paradigm prior to the main language processing task. This task was included as part of a larger lifespan study and will not be considered here. Secondly, a subset of participants completed two (rather than one) eyes-closed resting state EEG recording sessions both before and after the experiment: one in which they were instructed to relax and one in which they were asked to try to keep their mind blank. For the purposes of calculating resting-state individual difference metrics (IAF and 1/f slope), we used the eyes-closed recordings with the “relax” instructions, as these were comparable to the eyes-closed resting-state recordings with only a single session.

#### 3.1.6. EEG recording and preprocessing

The EEG was recorded from 64 electrodes mounted inside an elastic cap (actiCAP) using a Brain Products actiCHamp amplifier (Brain Products GmbH, Gilching, Germany). The electrooculogram (EOG) was recorded *via* electrodes placed at the outer canthi of both eyes as well as above and below the left eye. The EEG recording was sampled at 500 Hz and referenced to FCz.

Data preprocessing was undertaken as for Experiment 1 with the exception that, as a first step in the preprocessing procedure for Experiment 2, the data were converted to the brain imaging data structure for electroencephalography (EEG-BIDS; Pernet et al., 2019) using the MNE-BIDS Python package (Appelhoff et al., 2019).

##### 3.1.6.1. Resting-state EEG-based individual differences measures: Individual alpha frequency and aperiodic (1/f) activity

IAF and aperiodic slope estimates were calculated as for Experiment 1. Due to slightly differing electrode configurations, there were minor differences in the electrodes used for the IAF and aperiodic activity analyzes in this experiment. The electrodes used for IAF (peak alpha frequency) estimation were: P1, Pz, P2, PO3, POz, PO4, O1, O2. The electrodes used for aperiodic slope estimation were: F7, F3, Fz, F4, F8, FC5, FC1, FC2, FC6, T7, C3, Cz, C4, T8, CP5, CP1, CP2 CP6, P7, P3, Pz, P4, P8, PO9, O1, O2, PO10, AF7, AF8, F5, F1, F2, F6, FT7, FC3, FC4, FT8, C5, C1, C2, C6, TP7, CP3, CPz, CP4, TP8, P5, P1, P2, P6, PO7, PO3, POz, PO4, PO8.

#### 3.1.7. Data analysis

The data analysis was undertaken as for Experiment 1.

As our primary research question for Experiment 2 was whether it is possible to replicate the inter-individual difference effects observed in Experiment 1, we focus on the mixed model analyses examining 1/f slope, IAF and ID and how these modulate the effect of speaker-based surprisal across the course of the experiment.

### 3.2. Results

#### 3.2.1. Individual differences measures

Distributions of the (z-transformed) individual differences measures are shown in Supplementary Figure S2.

#### 3.2.2. EEG data

For the model including aperiodic slope, the top-level interactions involving Speaker-based Surprisal, Epoch and Slope were Prestimulus Amplitude x Speaker-based Surprisal x Epoch x Canonicity x Slope (Estimate = –0.0421, Std. Error = 0.0173, *z* = –2.44, *p* < 0.02) and Frequency x Speaker-based Surprisal x Epoch x Canonicity x Slope (Estimate = –0.0472, Std. Error = 0.0210, *z* = –2.24, *p* < 0.03).

For the model including IAF, the top-level interaction was Prestimulus Amplitude x Frequency x Speaker-based Surprisal x Epoch x IAF (Estimate = 0.0415, Std. Error = 0.0148, *z* = 2.80, *p* < 0.01); for the ID model, it was Prestimulus Amplitude x Frequency x Speaker-based Surprisal x Epoch x Canonicity x ID (Estimate = –0.0534, Std. Error = 0.0181, *z* = –2.95, *p* < 0.01). Model summaries are presented in Supplementary Tables S6–S8.

**Figure 7:**
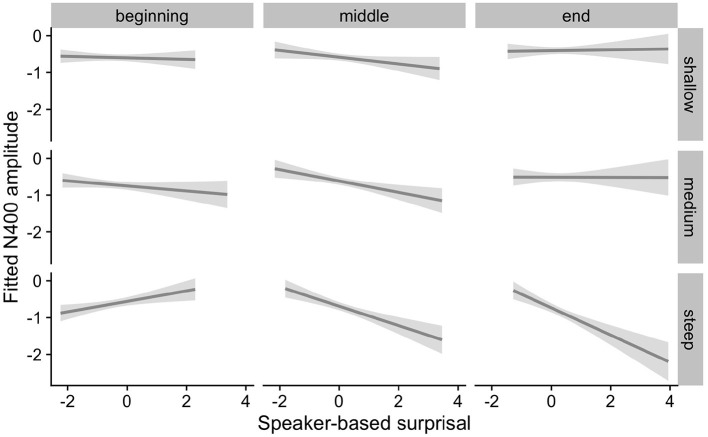
Effects of aperiodic (1/f) Slope on changes in the relationship between speaker-based surprisal (z-transformed) and N400 amplitude over the course of Experiment 2. The figure visualizes partial effects as calculated using the *remef* package, adjusted for Prestimulus Amplitude and Canonicity. Note that position in the experiment (operationalised *via* epoch in the statistical model) is trichotomised into beginning, middle and end for visualization purposes only; epoch was included in the model as a continuous predictor. The same holds for the individual differences variables, which are trichotomised for visualization purposes but were entered into the statistical models as a continuous predictors. Shaded areas indicate 95% confidence intervals.

**Figure 8:**
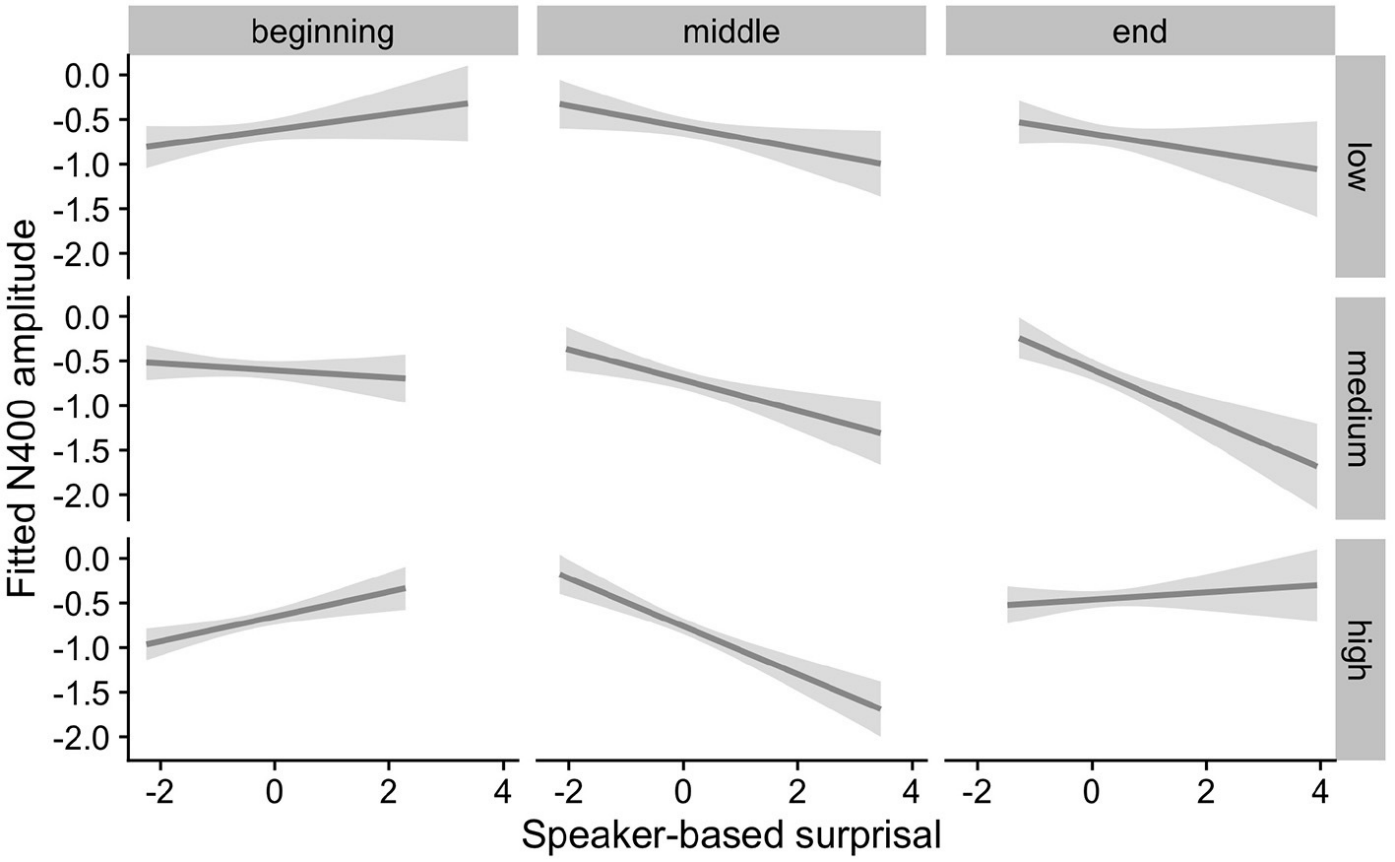
Effects of IAF on changes in the relationship between speaker-based surprisal (z-transformed) and N400 amplitude over the course of Experiment 2. The figure visualizes partial effects as calculated using the *remef* package, adjusted for Prestimulus Amplitude and Frequency. Note that position in the experiment (operationalised *via* epoch in the statistical model) is trichotomised into beginning, middle and end for visualization purposes only; epoch was included in the model as a continuous predictor. The same holds for the individual differences variables, which are trichotomised for visualization purposes but were entered into the statistical models as a continuous predictors. Shaded areas indicate 95% confidence intervals.

**Figure 9:**
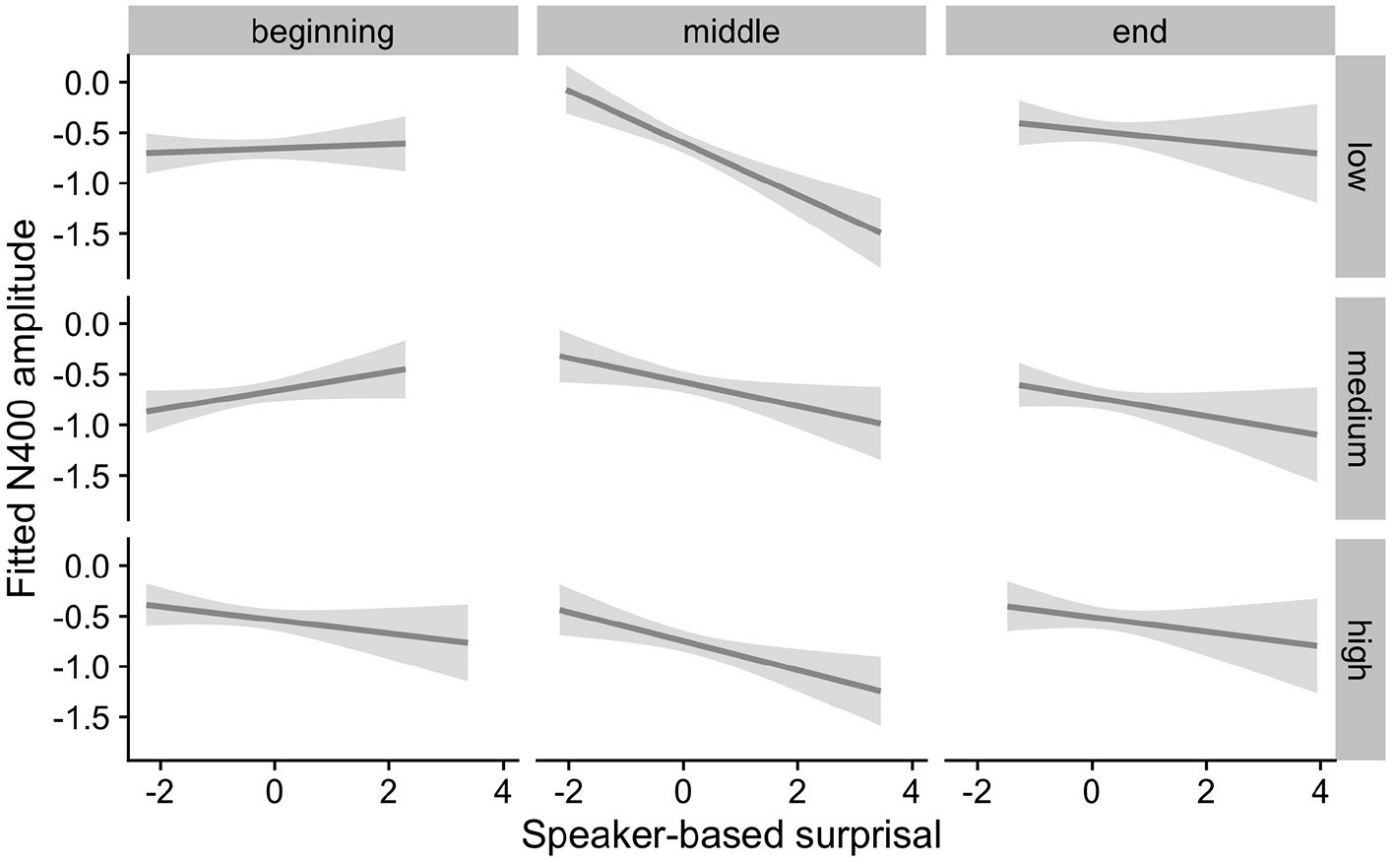
Effects of ID on changes in the relationship between speaker-based surprisal (z-transformed) and N400 amplitude over the course of Experiment 2. The figure visualizes partial effects as calculated using the *remef* package, adjusted for Prestimulus Amplitude, Frequency and Canonicity. Note that position in the experiment (operationalised *via* epoch in the statistical model) is trichotomised into beginning, middle and end for visualization purposes only; epoch was included in the model as a continuous predictor. The same holds for the individual differences variables, which are trichotomised for visualization purposes but were entered into the statistical models as a continuous predictors. Shaded areas indicate 95% confidence intervals.

The effects of interest are visualized in Figures 7–9. As for Experiment 1, we visualize partial effects of Speaker-based Surprisal x Epoch x Individual Differences Covariate of Interest for each model in turn in the following, adjusting for any additional moderating effects.

Overall, the results of Experiment 2 replicate the effects observed in Experiment 1. Individuals with a steep 1/f slope or a low IAF show more pronounced adaptation to speaker-based, intra-experimental probabilistic information over the course of the experiment in comparison to their counterparts with a shallow 1/f slope or a high IAF. By contrast, the pattern for ID is less clear.

### 3.3. Combined analysis of Experiments 1 and 2

Finally, we conducted a combined analysis of Experiments 1 and 2 in order to examine whether the inter-individual differences of interest would also be observable with a more substantial sample size (n=85). To this end, we again computed the three individual-differences models involving 1/f slope, IAF and ID using the same modeling approach as before. The only exception was the addition of a main effect of Experiment in the fixed effects in order to capture any intrinsic differences in EEG activity between the two experiments (e.g., due to the use of different amplifiers).

For the combined model including aperiodic slope, the top-level interactions involving Speaker-based Surprisal, Epoch and Slope were Prestimulus Amplitude x Frequency x Speaker-based Surprisal x Epoch x Slope (Estimate = 0.0352, Std. Error = 0.0113, *z* = 3.11, *p* < 0.01) and Prestimulus Amplitude x Speaker-based Surprisal x Epoch x Canonicity x Slope (Estimate = –0.0613, Std. Error = 0.0110, *z* = -5.58, *p* < 0.0001).

For the model including IAF, the top-level interactions of interest were Prestimulus Amplitude x Frequency x Speaker-based Surprisal x Epoch x IAF (Estimate = 0.0424, Std. Error = 0.0109, *z* = 3.88, *p* < 0.001) and Frequency x Speaker-based Surprisal x Epoch x Canonicity x IAF (Estimate = –0.0432, Std. Error = 0.0118, *z* = –3.65, *p* < 0.001). For the ID model, it was Prestimulus Amplitude x Frequency x Speaker-based Surprisal x Epoch x Canonicity x ID (Estimate = –0.0239, Std. Error = 0.0120, *z* = -1.99, *p* < 0.05). Model summaries are presented in Supplementary Tables S9–S11.

**Figure 10:**
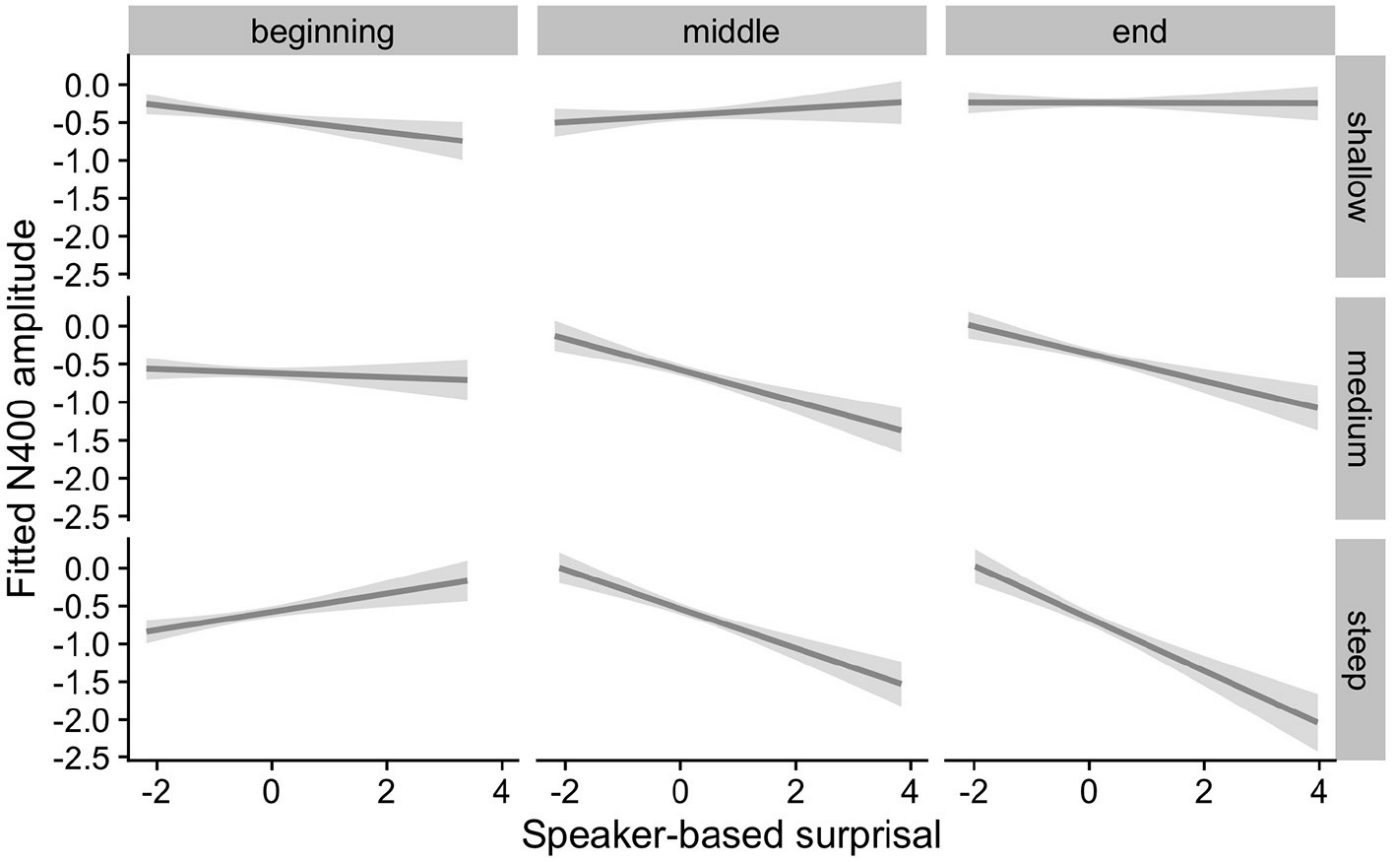
Effects of aperiodic (1/f) Slope on changes in the relationship between speaker-based surprisal (z-transformed) and N400 amplitude over the course of the experiment in the combined analysis of Experiments 1 and 2 (*n* = 85). The figure visualizes partial effects as calculated using the *remef* package, adjusted for Prestimulus Amplitude and Canonicity. Note that position in the experiment (operationalised *via* epoch in the statistical model) is trichotomised into beginning, middle and end for visualization purposes only; epoch was included in the model as a continuous predictor. The same holds for the individual differences variables, which are trichotomised for visualization purposes but were entered into the statistical models as a continuous predictors. Shaded areas indicate 95% confidence intervals.

**Figure 11:**
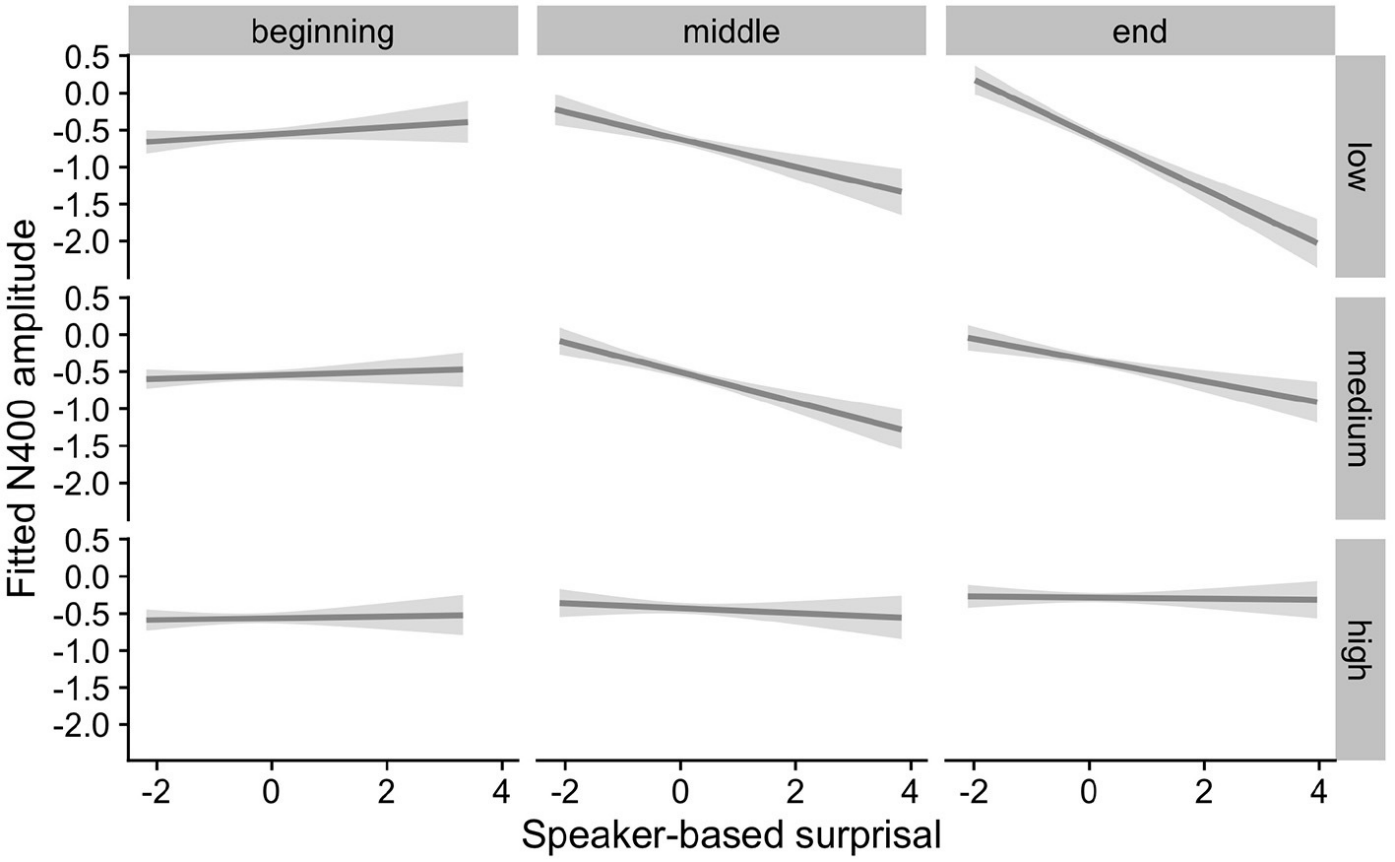
Effects of IAF on changes in the relationship between speaker-based surprisal (z-transformed) and N400 amplitude over the course of the experiment in the combined analysis of Experiments 1 and 2 (*n* = 85). The figure visualizes partial effects as calculated using the *remef* package, adjusted for Prestimulus Amplitude and Frequency. Note that position in the experiment (operationalised *via* epoch in the statistical model) is trichotomised into beginning, middle and end for visualization purposes only; epoch was included in the model as a continuous predictor. The same holds for the individual differences variables, which are trichotomised for visualization purposes but were entered into the statistical models as a continuous predictors. Shaded areas indicate 95% confidence intervals.

**Figure 12:**
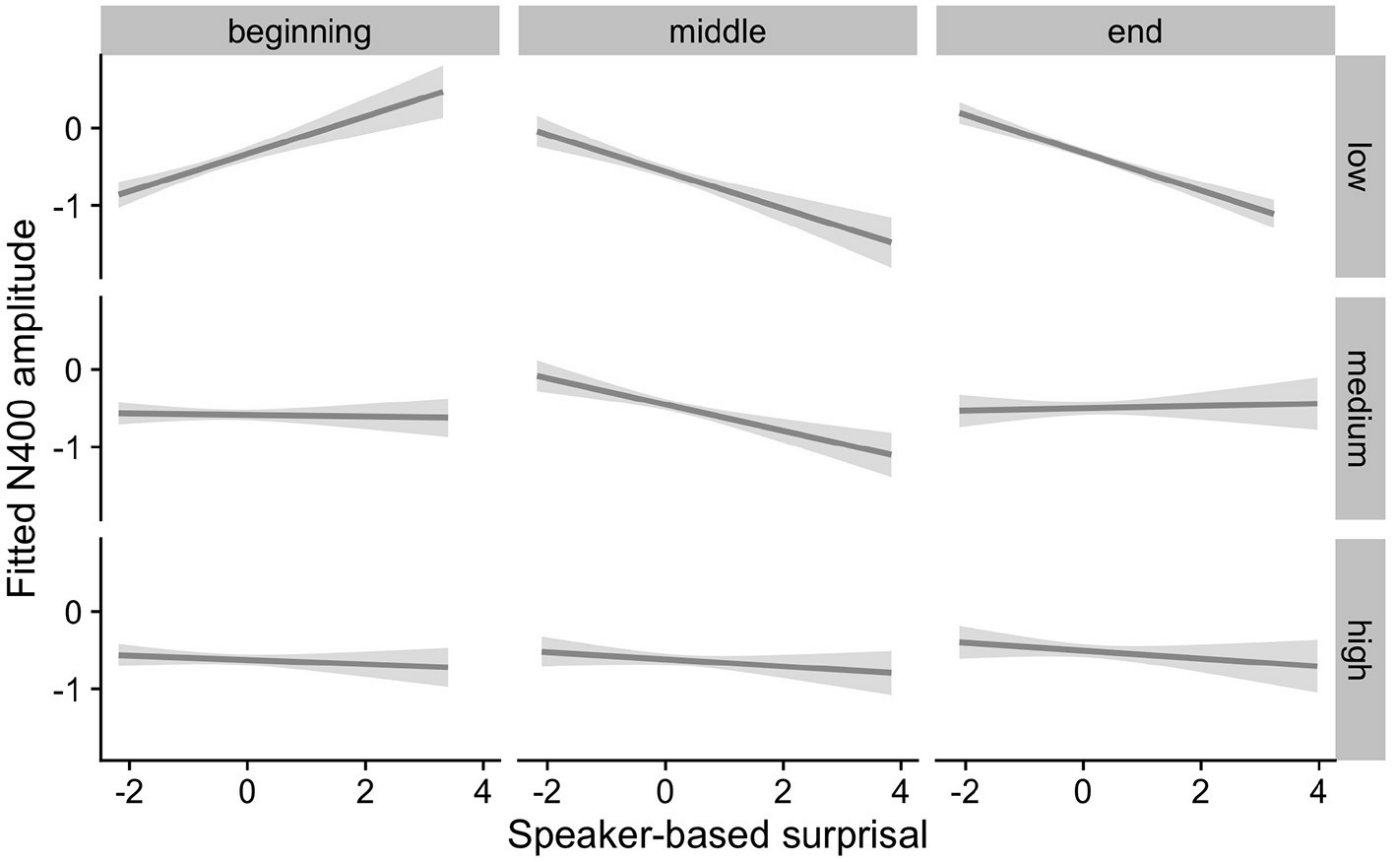
Effects of ID on changes in the relationship between speaker-based surprisal (z-transformed) and N400 amplitude over the course of the experiment in the combined analysis of Experiments 1 and 2 (*n* = 85). The figure visualizes partial effects as calculated using the *remef* package, adjusted for Prestimulus Amplitude, Frequency and Canonicity. Note that position in the experiment (operationalised *via* epoch in the statistical model) is trichotomised into beginning, middle and end for visualization purposes only; epoch was included in the model as a continuous predictor. The same holds for the individual differences variables, which are trichotomised for visualization purposes but were entered into the statistical models as a continuous predictors. Shaded areas indicate 95% confidence intervals.

The effects of interest are visualized in Figures 10–12. As for the analysis of Experiments 1 and 2, we visualize partial effects of Speaker-based Surprisal x Epoch x Individual Differences Covariate of Interest for each model in turn in the following, adjusting for any additional moderating effects.

### 3.4. Discussion

The results of Experiment 2 and the combined analysis of Experiments 1 and 2 broadly support the findings of Experiment 1. The findings for 1/f slope and IAF are highly compatible across all analyses: participants with a steep 1/f slope and those with a low IAF show a more substantial model adaptation to intra-experimental probabilistic information than those with a shallow 1/f slope or a high IAF. The findings for ID are not as clear for the individual analyses of Experiments 1 and 2; however, the combined analysis shows an emerging trend for increased model adaptation over the course of the experiment by individuals with a low ID.

## 4. General discussion

We have reported two ERP studies designed to investigate inter-individual differences in internal model updating during naturalistic language processing. By means of a novel measure of speaker-based surprisal for adjective orders, we examined the degree to which N400 responses track context-specific probabilistic information tied to the experimental environment. This measure, “speaker-based surprisal", reflects the predictability of adjective type for the second adjective in a two-adjective sequence given the type of the first adjective for a particular speaker. Adjective type was determined in a data-driven manner using a cluster-based analysis of semantic (word-vector-based) similarity between adjectives, and speaker-based probabilities were manipulated by having one speaker utter a higher percentage of expected orders and a second speaker utter a higher percentage of unexpected orders.

### 4.1. Individuals incrementally adapt their predictive language models to reflect current contextual information

The current findings present compelling evidence to suggest that individuals incrementally adapt their predictive language models to reflect current contextual information. In spite of only being exposed to new, intra-experimental adjective order regularities for a relatively short period of time, participants' N400 responses had attuned to this new information by the end of the experimental session. Strikingly, this rapid attunement occurred in spite of the wealth of linguistic experience that participants bring to the laboratory from their lifelong exposure to their native language. The importance of intra-experimental information vis-à-vis prior linguistic experience is further underscored by the observation that intra-experimental surprisal effects were aligned for canonical and non-canonical adjective orders by the end of the experiment. This suggests that experiment-specific adjective order probabilities eventually took on a higher weighting in shaping individuals' predictive models than their prior language experience.

Further attesting to the extremely fine-grained nature of the model adaptation process is the observation that N400 amplitude increasingly reflected intra-experimental adjective order surprisal, as calculated incrementally (i.e., on a trial-by-trial basis) for the experimental materials to which a participant had been exposed at each point in the experiment. Moreover, the adaptation took speaker-specific information into account (“speaker-based surprisal”). Previous studies have already demonstrated an adaptation of language comprehension processes to intra-experimental probabilities (Fine et al., 2013), including speaker-specific information (e.g., Kroczek and Gunter, 2017, 2021; Brothers et al., 2019). However, the present study is, to best of our knowledge, the first to demonstrate a gradual attunement to incremental, trial-by-trial fluctuations of intra-experimental, speaker-based surprisal over the course of an experiment.

When intra-experimental probabilities do not align with prior probabilities acquired through experience outside the laboratory, the precision of an individual's global language model is reduced. Model adaptation must thus take place to accommodate speaker-based, intra-experimental contingencies. These are increasingly incorporated into the listener's internal predictive model with increasing exposure to the experimental materials. The attunement of N400 amplitudes to speaker-based surprisal over the course of the experiment thus provides converging support for the proposal that N400 effects reflect precision-weighted prediction error signals (Bornkessel-Schlesewsky and Schlesewsky, 2019). As hypothesized by Bornkessel-Schlesewsky and Schlesewsky (2019), N400 effects thereby functionally mirror MMN effects as observed in auditory oddball paradigms designed to modulate predictive model precision (Todd et al., 2011, 2013, 2014). In these studies, the identity of standard and deviant tones within an auditory oddball paradigm was periodically changed, thus requiring an adaptation of the predictive model. Todd and colleagues observed increased MMN amplitudes within tone sequences that were presented for longer periods of time, i.e., when predictive models had sufficient time to stabilize and increase in precision. However, they also found a primacy effect such that MMN effects were larger for deviations from the tone that was initially established as the standard (Todd et al., 2011). This is indicative of an advantage for the first predictive model to be established and thus attests to the integration of new information with prior knowledge during the course of predictive model adaptation. We suggest that our results show a similar pattern: the observation of speaker-based surprisal effects at the level of adjective clusters demonstrates that intra-experimental contingencies were integrated with prior linguistic knowledge, since the clusters were derived using corpus-based word vectors. Participants were thus clearly still drawing on their prior knowledge of which adjectives tend to behave similarly, while at the same time adjusting their expectations based on the occurrence of these adjectives within the experiment.

### 4.2. Individual differences in predictive model adaptation

The fine-grained predictive model adaptation observed in the current study differed between individuals. In this regard, we had hypothesized that individuals with steeper 1/f slopes and individuals with higher ID would show a similar adaptation pattern on account of their strong predictive language models, and that this pattern would contrast with that observed for individuals with a higher IAF. Our results provided some converging support for these assumptions but also yielded some previously unexpected insights. Firstly, for 1/f slope and IAF, the directionality of the effects was the opposite of what we had expected: our results suggest a more pronounced adaptation for individuals with a steeper 1/f slope vs. less pronounced adaptation for individuals with a higher IAF. Secondly, the results for ID were less clear in the individual analyses of Experiments 1 and 2, but the combined analysis of both experiments revealed a trend for lower-ID individuals to show more rapid model adaptation, in line with our original hypothesis.

In the following, we discuss 1/f slope, IAF and ID in turn.

#### 4.2.1. Individuals with a steeper aperiodic (1/f) slope show more pronounced effects of model adaptation than those with a shallower aperiodic slope

Participants with a steeper aperiodic (1/f) slope showed a more substantial N400 attunement to speaker-based surprisal over the course of the experiment than their counterparts with a shallower aperiodic slope. This result supports and extends the findings by Dave et al. (2018) that individuals with a steep 1/f slope showed more pronounced prediction-related N400 effects than individuals with a shallow 1/f slope. Dave and colleagues proposed that individuals with low neural noise, as reflected in a steeper 1/f slope, show enhanced prediction (i.e., their study showed a relationship between 1/f slope and N400 effects marking successful vs. unsuccessful lexical prediction). While we had originally hypothesized that this might correlate with a reduced degree of adaptation to intra-experimental contigencies, our findings suggest that, to the contrary, enhanced prediction may in fact be related to an individual's ability to flexibly adapt their neural predictive coding infrastructure to current environmental and task conditions.^2^

This assumption can be linked to the notion that steeper 1/f slopes are indicative of lower levels of neural noise. It is proposed that steeper 1/f slopes in both intracranial and scalp EEG reflect more synchronous neural firing and concomitantly lower rates of aberrant firing or random background activity (for a review of the physiological mechanisms and modeling work that supports this claim, see Voytek and Knight, 2015). The higher signal-to-noise ratio associated with this more synchronous activity can be viewed as reflecting lower neural noise (Hong and Rebec, 2012)^3^. An increase of random neural background activity in aging (increased neural noise) goes hand in hand with increased variability and slowing of neural and behavioral responses to external stimuli (Hong and Rebec, 2012) as well as with a flattening of 1/f slope (Voytek et al., 2015). For example, Tran et al. (2020) observed that increased resting-state neural noise, as reflected in a flatter 1/f slope, in older adults correlated with increased variability of stimulus-related neurophysiological responses (peak alpha inter-trial coherence, ITC) in a visual discrimination task. In relation to predictive coding, lower neural noise possibly allows for a more dynamic and efficient adaptation of task- and context-related neural networks in accordance with current task demands, thus facilitating accurate and context-appropriate predictions.

Pertermann et al. (2019b) recently suggested that there is a relationship between neural noise as indexed by 1/f and neural gain control *via* the noradrenergic system. Release of noradrenaline from the brainstem locus coeruleus leads to increased excitatory and decreased inhibitory responses to a stimulus of interest, thus resulting in stronger stimulus discriminability and a more binary response function (i.e., stronger neural gain, Aston-Jones and Cohen, 2005). In their study, Pertermann et al. (2019b) observed a correlation between 1/f slope and pupil dilation—an index of noradrenergic system activation—in a go/no-go task and specifically for no-go trials requiring response inhibition.

The potential link between lower neural noise and higher neural gain suggests that individuals with a steeper aperiodic slope may be more effective in discriminating between relevant and irrelevant information for the flexible adaptation of their predictive models to the current context. This aligns with an active inference perspective on attention, according to which attention is preferentially allocated toward sensory evidence with a high precision (Parr and Friston, 2017). By optimizing the allocation of attention toward salient/task-relevant information, this could lead to a more rapid establishment of higher-precision models by individuals with a steeper 1/f slope—or, perhaps more precisely, models in which precision is appropriately weighted in light of prior evidence.

#### 4.2.2. Stronger model adaptation for individuals with lower individual alpha frequency

Turning now to IAF, it initially appears somewhat counterintuitive that individuals with a higher IAF show less predictive model adaptation than individuals with a lower IAF. After all, higher IAF correlates with faster processing cycles (Cecere et al., 2015; Samaha and Postle, 2015) and previous findings suggest that older adults with a high IAF show a higher propensity to reanalyze ambiguous (“garden path") sentences when it becomes apparent that the reading initially adopted was incorrect (Kurthen et al., 2020). On the basis of these previous observations, we had thus hypothesized that high-IAF individuals would show a higher propensity for predictive model adaptation than low-IAF individuals. Upon closer consideration, however, the present study differed from the above-cited studies in several important respects. Firstly, in the study by Kurthen et al. (2020), reanalysis did not require an adaptation of the predictive model but rather the correction of a previous processing decision within the bounds of the current model's strategy space. By contrast, the adaptive demands of the present study required participants to learn new, intra-experimental probabilities associated with each speaker and adapt their predictive models to these new contingencies. Secondly, the time frames relevant for these adaptive learning processes were substantially longer than the perceptual windows of interest in the studies by Cecere et al. (2015) and Samaha and Postle (2015), as participants were required to learn two-adjective sequencing regularities over the course of an experimental session. Previous work on the localization of targets moving in space revealed an advantage for individuals with a lower IAF (Howard et al., 2017), with the authors suggesting that this result could be due to the longer timescales involved in the task (movement was between 2 and 4 s in length) in comparison to the transient stimuli used, for example, by Samaha and Postle (2015). In the language domain, Nalaye et al. (2022) recently found that lower-IAF individuals outperformed their higher-IAF counterparts when learning a modified miniature language based on Mandarin Chinese. Akin to the study by Howard et al. (2017), this paradigm involved learning regularities on timescales of multiple seconds. In the present study, lower-IAF individuals may have likewise been better able to adapt their predictive models to the intra-experimental probabilities that unfolded over multiple seconds (intra-stimulus) and minutes (inter-stimulus). However, this explanation remains tentative at present and requires more systematic examination in future research.

#### 4.2.3. A more complex relationship between model adaptation and idea density

As ID measures the efficiency of linguistic encoding (Cheung and Kemper, 1992; Kemper et al., 2001b; Iacono et al., 2009; Engelman et al., 2010; Farias et al., 2012), we examined it as a proxy for the quality of an individual's language model. We thus hypothesized that individuals with lower ID and, hence, a lower quality language model, would show a faster adaptation to new linguistic information. While the results of Experiments 1 and 2 both showed a less clear pattern for ID in comparison to 1/f slope and IAF, the combined analysis of the two experiments does provide some converging evidence for the hypothesis that lower-ID individuals adapted their language models more substantially to the intra-experimental contingencies presented to them.

Low ID in young adulthood is a risk factor for cognitive decline and dementia in old age (Snowdon et al., 1996; Kemper et al., 2001a) and has been suggested to reflect “suboptimal neurocognitive development" (Kemper et al., 2001a, p.602). The notion that lower-ID individuals show a more flexible adaptation of their internal predictive models to the current environment may thus, at a first glance, appear somewhat counterintuitive. Note, however, that faster adaptation in the present study should not necessarily be considered a superior processing strategy. After all, high adaptability means that individuals adjusted expectations accrued through a lifetime of language experience to speaker-specific patterns encountered within a brief experimental session. This could, at least under certain circumstances, lead to the type of “overfitting” of internal predictive models that may be problematic for cognitive performance in older adulthood (Moran et al., 2014).

To better examine the utility of a rapid adaptation strategy, future research could consider model adaptation in different reward contexts, i.e., comparing circumstances where high model malleability is useful to those where it is detrimental to optimal performance. This could yield further insights on calibrated model adaptation, in which the strong prior evidence provided by a high-quality language model is weighed against the increasing quantity of incoming evidence which contradicts the prior model. In addition, the role of domain specificity requires further consideration: of our three individual differences measures of interest, only ID was directly related to the domain under consideration (language), while the other two can be considered to reflect more general characteristics of neural information processing. Future research will need to examine the role of such purported domain-specific vs. domain-general influences in more detail.

Such considerations also reflect a limitation of the current study, namely that possible interactions between individual differences measures were not considered. These are, in our view, outside of the scope of what is already a highly complex pattern of results in a new domain of investigation. However, if our interpretation of the present findings is correct, future studies should be able to further illuminate the mechanics of individuals' model adaptation by taking into account the interplay of the various individual differences metrics examined here.

### 4.3. Implications for predictive coding in language and beyond

Our results demonstrate that predictive processing during language comprehension adapts flexibly to current contextual and environmental demands, involving both intrinsic linguistic properties (adjective type) as well as communicative aspects (identity of the speaker). They thus extend previous work linking N400 responses to surprisal (e.g., Frank et al., 2015; Frank and Willems, 2017) by demonstrating that corpus-based surprisal may need to be complemented by surprisal metrics that are more closely aligned to the experimental context. To further understand the implications of our findings for predictive coding in language, future research should examine the persistence of predictive model adaptations. It appears unlikely that a single session of exposure to new grammatical or communicative regularities would lead to a permanent adaptation of linguistic models. The application of adapted models to future situations could, however, be governed by cognitive control processes such as those proposed in hierarchical models of cognitive control (e.g., Koechlin and Summerfield, 2007). Here, contextual or episodic information provides control cues to override prepotent stimulus-response mappings and instantiate new mappings for the duration of the appropriate context's or episode's presence. Within the context of the present study, speaker identity could have functioned as one such control cue—in addition to the broader contextual cue of undertaking a language processing task in a laboratory. Participants with a steeper 1/f slope and lower neural noise may be more adept at using such control cues to flexibly switch between alternate predictive models (cf. the association between 1/f neural noise and cognitive control in non-neurotypical populations such as children with ADHD; Pertermann et al., 2019a; Robertson et al., 2019; Ostlund et al., 2021).

A more comprehensive understanding of language processing in contextually rich, naturalistic settings could thus be facilitated by a closer examination of the interplay between predictive coding and cognitive control. Alternatively, cognitive control mechanisms could even be couched within a predictive coding architecture, as proposed by the Hierarchical Error Representation (HER) framework. The HER, which is able to account for a wide range of cognitive control-related findings including hierarchical aspects of cognitive control, posits that “a major function of prefrontal cortex is learning to predict likely prediction errors” (Alexander and Brown, 2018, p.2).

Such an approach could have far-reaching implications for language, including in helping to link linguistic phenomena across different timescales: from processing mechanisms at the scale of tens or hundreds of milliseconds to language change. We have previously suggested that precision-weighted prediction error signals could provide an “early warning signal” for impending language change (Bornkessel-Schlesewsky et al., 2020). Specifically, based on findings from Icelandic, we proposed that reduced N400 effects to a construction that is incompatible with the current prescriptive grammar signal lower predictive precision and, hence, a possible propensity for change. The present findings provide converging support for the very early stages of this proposed process by showing how a loss of precision for a prior linguistic model can lead to rapid model adaptation in accordance with current environmental contingencies. They further suggest that the temporal trajectories for model adaptation differ between individuals, with early adopters being characterized by lower neural noise (steeper aperiodic slope), lower Individual Alpha Frequency and, possibly, lower Idea Density.

## Data availability statement

The datasets presented in this study can be found in online repositories. The names of the repository/repositories and accession number(s) can be found below: https://osf.io/32amz/.

## Ethics statement

The studies involving human participants were reviewed and approved by University of South Australia's Human Research Ethics Committee. The participants provided their written informed consent to participate in this study.

## Author contributions

IB-S, IS, CH, and EW prepared the experiments. IS, CH, and EW collected the data. IB-S and RK performed the data analysis. IB-S wrote the first draft of the manuscript. All authors contributed to conception and design of the study, manuscript revision, and approved the submitted version.

## Funding

The research reported here was funded by an Australian Research Council Future Fellowship awarded to IB-S (FT160100437). AC acknowledges the support of the Three Springs Foundation.

## Conflict of interest

Author PA was employed by Beacon Biosignals, Boston. The remaining authors declare that the research was conducted in the absence of any commercial or financial relationships that could be construed as a potential conflict of interest.

## Publisher's note

All claims expressed in this article are solely those of the authors and do not necessarily represent those of their affiliated organizations, or those of the publisher, the editors and the reviewers. Any product that may be evaluated in this article, or claim that may be made by its manufacturer, is not guaranteed or endorsed by the publisher.

## Footnotes

^1^For an alternative proposal, see, for example, Spratling (2008).

^2^It is worth noting in this context that Dave et al. (2018) examined on-task 1/f activity during their sentence comprehension tasks, while we examined resting-state 1/f activity in the present study. Some recent research suggests that 1/f slope can be linked to global states of consciousness and arousal (Lendner et al., 2020), which could affect predictive model updating through improved attentional regulation and, hence, increased sensitivity to both prediction errors and contextual states. Future research will need to further examine the relationship between resting and on-task 1/f.

^3^A complementary perspective on the physiological underpinnings of the 1/f slope is that it indexes the balance between excitatory and inhibitory activity: a flatter slope correlates with more stochastic excitatory firing, which is consistent with reduced inhibitory firing in aging (Gao et al., 2017).
