# Supplementary material for: Rapid adaptation of predictive models during language comprehension: Aperiodic EEG slope, individual alpha frequency and idea density modulate individual differences in real-time model updating
Source: Front Psychol. 2022 Aug 26;13:817516. doi: 10.3389/fpsyg.2022.817516 (PMC9461998; doi:10.3389/fpsyg.2022.817516)
Supplement: Supplementary file 1 [file Data_Sheet_1.PDF]

Supplementary Materials for the paper "Rapid adaptation of predictive models during language comprehension: aperiodic EEG slope, individual alpha frequency and idea density modulate individual differences in real-time model updating"

1 PROBABILISTIC PREDICTABILITY IN LANGUAGE

Examples (1) and (2) present a selected (and non-exhaustive) list of information sources that can give rise to probabilistic predictability in language.

1. **"Global" predictabilities**
   a. Category
   Example: A determiner (e.g. "the") will, at some point, be followed by a noun (e.g. "hamster").[1]
   b. Subcategorisation
   Example: A transitive verb (e.g. "to run over") requires an object (e.g. "a hamster").
   c. Semantic
   Example 1, semantic fields: The words "apple" and "eat" have a higher likelihood of occurring together in a sentence than the words "apple" and "sneeze"; this is captured by techniques such as latent semantic analysis (LSA; Landauer et al., 1998).
   Example 2, semantic dependencies: "The opposite of black" leads to a high predictability for "white".
   d. Case or role dependencies
   Example: An object at the beginning of the sentence leads to the prediction that a subject will follow at some later point (e.g. Gibson, 1998). This dependency may be signalled via morphological case marking in some languages (e.g. German: Bornkessel et al., 2004) and even appears to hold in languages permitting subject drop (e.g. Japanese: Wolff et al., 2008).
2. **"Local" predictabilities**
   a. Transition probabilities
   Example: Bigram or trigram frequencies, which reflect how often groups of two (e.g. "small hamster") or three ("the small hamster") words occur in sequence (more generally: n-grams to refer to sequences of n words).[2]
   b. The global likelihood of encountering an individual word
   A word's frequency of occurrence can be viewed as a global prior on the likelihood of encountering it.

[1] Note that the precedence information implied here – that the noun follows the determiner – is language specific. While determiners precede nouns in English and other Western European languages, this is not universal across the languages of the world.

[2] Note that, while we refer to lexical n-grams (i.e. sequences of individual words) here, n-grams can also be calculated using other features such as word category.

2 SUPPLEMENTARY MATERIALS FOR EXPERIMENT 1

2.1 Adjective clusters

Adjective clusters for Experiment 1 (for the clustering procedure, see the main text):

1. angry anxious busy confused cracked dirty dreaded eager empty endless evil flustered foolish frail good hard harmless hostile hyped ignored injured jetlagged lazy little lonely loud lucky morning naughty nervous obsessed odd old pleased poor random ripped ripping silly slow startled stingy strange stumbling stupid sudden tired tiring tricky tugging upset weird wicked
2. cold cramped crowded dark dusty flushed freezing grassy hot humid muddy pouring rainy rolling soft steaming sticky sweaty thick warm wet
3. black blue brown coloured gold green grey orange pale pink purple red silver speckled white yellow
4. brave bright calm cheerful clean cool festive fierce friendly gentle gorgeous graceful handsome happy healthy intense joyful lovely loving moody nice offbeat peaceful placid playful quiet rich ripe soothing spacious stark sweet tasty trendy vibrant vivid wild
5. abrupt active aged big clear constant crystal cut daily deep engaged English famous fine first fit gifted grand great heavy high huge human large long main massive metal narrow new ornate plain polished quick raised remote renowned round sacred sharp short skilled small square steep strict strong thorough three tiled timber unused varied winning wooden worn young
6. bland blonde brunette bushy buttered cute fancy fat fluffy ginger heeled hunched juicy nimble pricy rusty scented shiny skinny slender slim smooth sparkly squishy tall thin tight tiny ugly wiggling

2.2 Figures

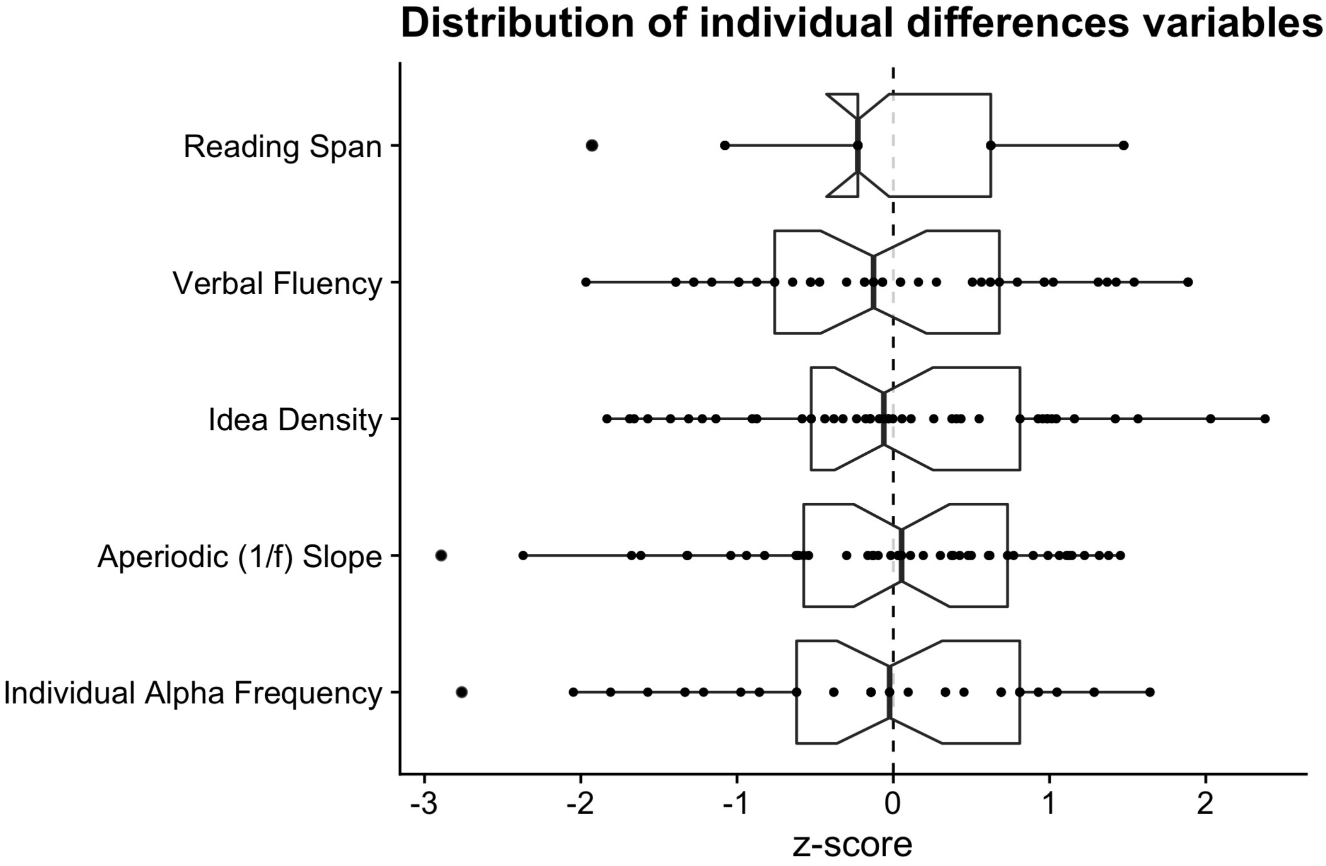

Figure S1. Distributions of individual differences measures in Experiment 1. Note that, in addition to the individual differences variables of primary interest for the present study, we also show the distributions for Reading Span and Verbal Fluency, as these have been examined most extensively in the previous literature on language processing.

2.3 Tables

The following tables provide full model summaries for the linear mixed effects models described in the main text of the paper. Note that the tables only report significant main effects and interactions in order to improve readability. Effects discussed in the main text are highlighted in bold. For full model summaries, please see the OSF repository for this paper (see Data Availability statement in the main text).

For all of the following tables, effects are abbreviated as follows: ps - prestimulus amplitude; fq - unigram frequency; ss - speaker-based surprisal; sg – global surprisal; ep - epoch; cn - canonicity; id - ID; paf – peak alpha frequency (IAF); slope – aperiodic (1/f) slope; ps2 - quadratic prestimulus amplitude; ss2 - quadratic speaker-based surprisal; ss3 - cubic speaker-based surprisal. Columns Est., SE, z and p provide the estimate, standard error, z-statistic and p-value for each of the fixed effects in the model. The σ columns include the variance components of the model, i.e. random intercepts and slopes by item, subject and channel.

Table S1: **Model summary for the sanity check model in Experiment 1**

	Est.	SE	z	p	σ_{Item}	σ_{Subj}	σ_{Channel}
(Intercept)	-0.4986	0.0745	-6.69	<1e-10	0.4943	0.2565	0.1511
ps	2.8913	0.1367	21.16	<1e-98	0.4325	0.6543	0.3403
**ps x fq x sg**	0.0497	0.0203	2.45	0.0143			
Residual	4.1769						

Table S2: **Model summary for the best-fitting model without individual differences in Experiment 1**

	Est.	SE	z	p	σ_{Item}	σ_{Subj}	$\sigma_{Channel}$
(Intercept)	-0.4134	0.0792	-5.22	$<$1e-06	0.5912	0.2681	0.1581
ps	2.8646	0.1368	20.94	$<$1e-96	0.4341	0.6621	0.3349
ss2	-0.0855	0.0240	-3.57	0.0004			
ss3	0.0279	0.0118	2.36	0.0182			
ps x ss	-0.0417	0.0197	-2.11	0.0348			
fq x ss	0.1156	0.0371	3.12	0.0018			
ps x ep	0.1368	0.0178	7.68	$<$1e-13			
fq x ep	0.1522	0.0289	5.26	$<$1e-06			
ep x cn	-0.1298	0.0210	-6.19	$<$1e-09			
fq x ss x cn	0.2041	0.0336	6.08	$<$1e-08			
ss x ep x cn	-0.0553	0.0224	-2.48	0.0133			
ps x fq x ss x ep	0.0643	0.0185	3.48	0.0005			
ps x fq x ss x cn	0.0692	0.0189	3.65	0.0003			
ps x fq x ep x cn	0.0420	0.0187	2.24	0.0251			
**ps x ss x ep x cn**	-0.0693	0.0173	-4.01	$<$1e-04			
Residual	4.1037						

Table S3: **Model summary for the best-fitting model including 1/f slope in Experiment 1**

	Est.	SE	z	p	σ_{Item}	σ_{Subj}	σ_{Channel}
ps	4.5303	0.4812	9.41	<1e-20	0.4309	0.5818	0.3366
ss2	-0.0870	0.0245	-3.55	0.0004			
ss3	0.0316	0.0122	2.60	0.0094			
ps x fq	-0.2073	0.0976	-2.12	0.0338			
ps x ss	-0.2335	0.0983	-2.37	0.0176			
fq x ss	-0.2385	0.1050	-2.27	0.0232			
ps x ep	0.2473	0.0997	2.48	0.0131			
ss x ep	0.3669	0.0973	3.77	0.0002			
ss x cn	0.3473	0.0997	3.48	0.0005			
ep x cn	-0.2618	0.0989	-2.65	0.0081			
ps x slope	1.3042	0.3664	3.56	0.0004			
ps x ss x ep	-0.2690	0.0980	-2.75	0.0060			
ps x ss x cn	-0.3023	0.0976	-3.10	0.0019			
ps x ep x cn	-0.2755	0.1001	-2.75	0.0059			
fq x ep x cn	0.3114	0.1024	3.04	0.0024			
ss x ep x cn	-0.3306	0.0961	-3.44	0.0006			
ps x ss x slope	-0.1509	0.0753	-2.00	0.0451			
fq x ss x slope	-0.2847	0.0790	-3.60	0.0003			
ss x ep x slope	0.3440	0.0748	4.60	<1e-05			
ss x cn x slope	0.2374	0.0770	3.08	0.0021			
ps x fq x ss x cn	-0.2358	0.0987	-2.39	0.0169			
ps x ss x ep x cn	-0.2954	0.0978	-3.02	0.0025			
ps x ss x ep x slope	-0.2127	0.0749	-2.84	0.0045			
ps x ss x cn x slope	-0.2572	0.0750	-3.43	0.0006			
fq x ss x cn x slope	-0.2163	0.0781	-2.77	0.0056			
ps x ep x cn x slope	-0.2438	0.0770	-3.17	0.0015			
fq x ep x cn x slope	0.2094	0.0796	2.63	0.0085			
ss x ep x cn x slope	-0.2232	0.0749	-2.98	0.0029			
ps x fq x ss x ep x cn	0.2127	0.1030	2.06	0.0389			
ps x fq x ss x cn x slope	-0.2271	0.0747	-3.04	0.0024			
ps x ss x ep x cn x slope	-0.1709	0.0749	-2.28	0.0226			
**ps x fq x ss x ep x cn x slope**	0.1531	0.0780	1.96	0.0495			
Residual	4.0735						

Table S4: **Model summary for the best-fitting model including IAF in Experiment 1**

	Est.	SE	z	p	σ_{Item}	σ_{Subj}	$\sigma_{Channel}$
ps	2.1599	0.9653	2.24	0.0253	0.4403	0.6630	0.3333
ss	-1.4903	0.4211	-3.54	0.0004	0.8731	0.2635	
cn	0.7686	0.3516	2.19	0.0288	0.5592	0.2182	
ss2	-0.0824	0.0248	-3.32	0.0009			
ss3	0.0301	0.0123	2.44	0.0146			
ps x fq	0.3539	0.1456	2.43	0.0151			
fq x ss	0.6247	0.1642	3.81	0.0001			
ps x cn	0.4799	0.1420	3.38	0.0007			
ep x cn	-0.4439	0.1642	-2.70	0.0069			
ss x paf	0.1350	0.0416	3.24	0.0012			
fq x ss x cn	0.8841	0.1647	5.37	<1e-07			
ps x fq x paf	-0.0384	0.0148	-2.60	0.0094			
fq x ss x paf	-0.0498	0.0161	-3.09	0.0020			
ps x cn x paf	-0.0465	0.0144	-3.22	0.0013			
ps x fq x ss x ep	0.0622	0.0185	3.36	0.0008			
ps x fq x ss x cn	0.0648	0.0190	3.40	0.0007			
ps x fq x ep x cn	0.0410	0.0188	2.18	0.0291			
ps x ss x ep x cn	-0.0716	0.0174	-4.12	<1e-04			
fq x ss x ep x cn	0.5466	0.1721	3.18	0.0015			
fq x ss x cn x paf	-0.0661	0.0163	-4.06	<1e-04			
**fq x ss x ep x cn x paf**	-0.0505	0.0172	-2.94	0.0033			
Residual	4.0833						

Table S5: **Model summary for the best-fitting model including ID in Experiment 1**

	Est.	SE	z	p	σ_{Item}	σ_{Subj}	σ_{Channel}
(Intercept)	-0.4170	0.0792	-5.27	<1e-06	0.5875	0.2687	0.1593
ps	2.8587	0.1364	20.96	<1e-96	0.4316	0.6607	0.3337
ss2	-0.0894	0.0241	-3.71	0.0002			
ss3	0.0298	0.0120	2.50	0.0125			
ps x ss	-0.0514	0.0198	-2.60	0.0094			
fq x ss	0.1282	0.0373	3.44	0.0006			
ps x ep	0.1434	0.0178	8.05	<1e-15			
fq x ep	0.1473	0.0292	5.05	<1e-06			
ss x ep	-0.0566	0.0274	-2.07	0.0388			
ep x cn	-0.1185	0.0211	-5.62	<1e-07			
fq x ss x cn	0.2091	0.0339	6.18	<1e-09			
ss x ep x cn	-0.0553	0.0224	-2.47	0.0135			
ps x fq x id	-0.0468	0.0164	-2.86	0.0043			
ps x ss x id	0.0474	0.0163	2.90	0.0037			
fq x ss x id	0.0726	0.0178	4.07	<1e-04			
fq x ep x id	-0.0954	0.0177	-5.39	<1e-07			
ss x ep x id	0.0404	0.0170	2.38	0.0174			
ps x cn x id	-0.0583	0.0167	-3.50	0.0005			
fq x cn x id	0.0574	0.0200	2.87	0.0041			
ep x cn x id	0.0827	0.0176	4.70	<1e-05			
ps x fq x ss x ep	0.0627	0.0186	3.38	0.0007			
ps x fq x ss x cn	0.0739	0.0190	3.89	0.0001			
ps x fq x ep x cn	0.0443	0.0188	2.36	0.0182			
ps x ss x ep x cn	-0.0630	0.0173	-3.64	0.0003			
ps x fq x ep x id	-0.0531	0.0163	-3.26	0.0011			
fq x ss x cn x id	0.0373	0.0179	2.08	0.0374			
fq x ep x cn x id	-0.0681	0.0185	-3.69	0.0002			
ps x fq x ss x cn x id	-0.0555	0.0158	-3.50	0.0005			
**ps x ss x ep x cn x id**	0.0821	0.0163	5.03	<1e-06			
Residual	4.0770						

3 SUPPLEMENTARY MATERIALS FOR EXPERIMENT 2

3.1 Adjective clusters

Adjective clusters for Experiment 2 (for the clustering procedure, see the main text; Methods section for Experiment 1):

1. active aged aggressive attentive big brief challenging colossal competitive consistent covert daily daring disabling disastrous dramatic eager earnest elderly elite energetic English engrossed enormous enticing esteemed exacting exceptional expensive familiar famous favourite feared fierce first friendly gifted grand great grounding high huge human hyper impressive inviting junior keen large lavish local main massive modern neglected new novice old ornate outdoor outspoken overwhelmed own personal pleased popular precious prestigious prompt raised recurrent regional remote renowned revered rival round rural rushed sensitive simple skilled small strict strong succinct thorough towering usual varied vast visiting weekly winning wise young younger youngest zealous
2. black blue bronze brown buttered coloured crystal finned flushed giant ginger gold green grey metal orange pink purple red ripped rusty scented shaded shiny silver square tiled tin tiny white wooden worn yellow
3. adorable beautiful bland bohemian bright calm carefree charming chatty cheeky cheerful chic colourful cool cosy cute dazzling delicious delightful festive fine fizzy fluffy fragrant fun gentle good haunting healthy joyful juicy lovely loving moody nice offbeat opulent placid playful polished pricy pristine quiet radiant ripe sociable soothing spacious sparkly squishy stylish sunny sweet tacky tasty tidy trendy vibrant warm
4. abrupt arduous bare bouncing chaotic clean clear cold constant cracked cramped crowded cut dark deep difficult dreary dusty endless fit flat freezing harsh heavy hectic hot humid intense leaky lengthy long loud mild morning muddy narrow noisy painful plain pouring protracted quick rainy relentless ripping rocky rolling rough short slow sluggish soft sprawling stark steaming steep sticky stressful sudden sweaty tedious tight tiring tough trembling wet winding
5. alarmed anxious awkward blatant bloody boring busy careless cheap clueless clumsy conceited confused dirty dishevelled disruptive distracted dreaded dreadful drunken eccentric enraged evil flustered foolish frail geriatric ghastly graceless grubby hapless happy harmless hostile idle inattentive inept inexperienced injured innocent insolent jetlagged jittery kind laughing lazy limping little lonely lucky mediocre miserable naive naughty negligent nervous odd panicked poor rebellious reckless restless sad scared sickly silly sneaky startled stingy strange stupid tired tugging ugly unaware unfortunate unlucky unprincipled untrained upset violent wandering weary weird wiggling wrongful
6. agile athletic bearded blonde brunette burly bushy fat freckled graceful grassy lanky nimble scruffy shy skinny slender slim smooth stealthy stocky tall tanned thick thin upright wiry

3.2 Figures

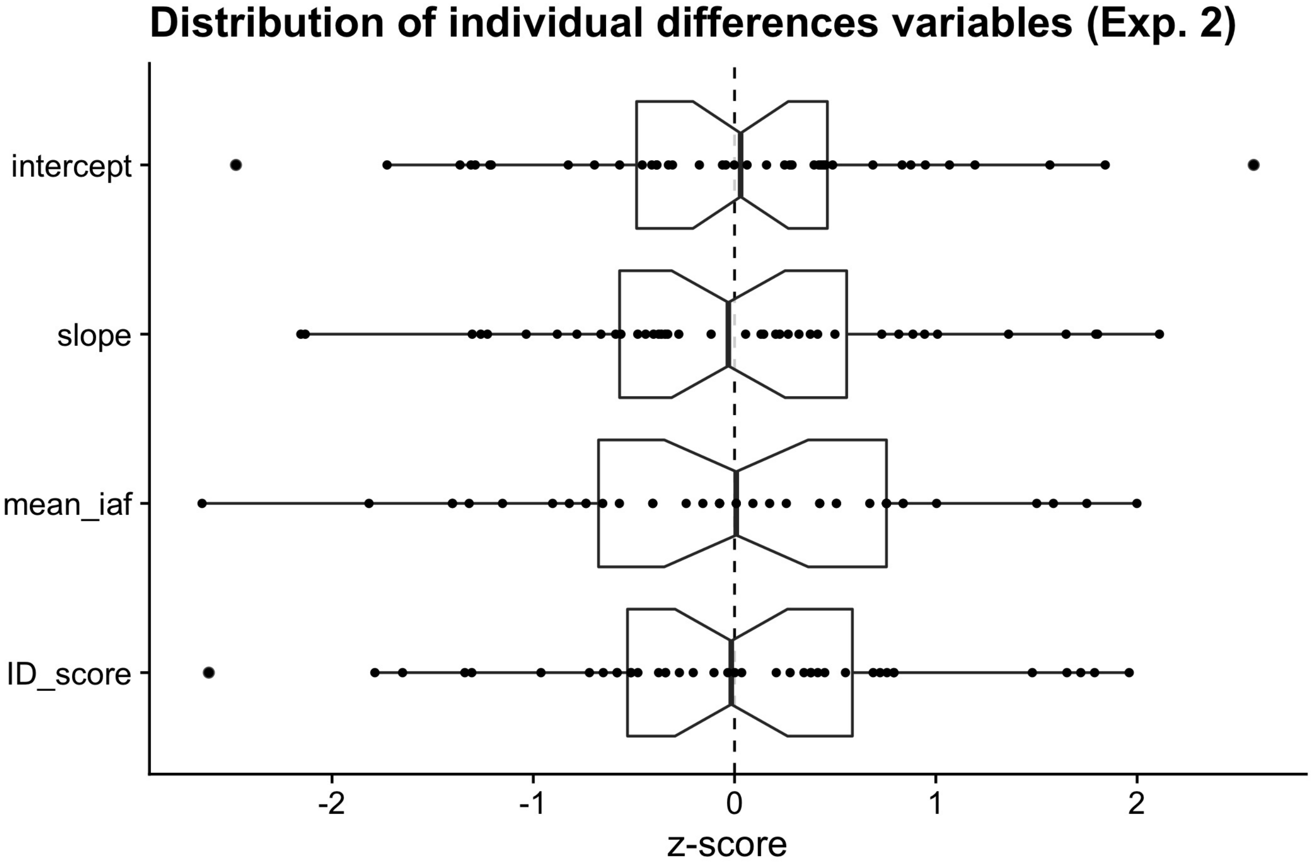

Figure S2. Distributions of individual differences measures in Experiment 2.

3.3 Tables

The following tables provide full model summaries for the linear mixed effects models described in the main text of the paper. Note that the tables only report significant main effects and interactions in order to improve readability. Effects discussed in the main text are highlighted in bold. For full model summaries, please see the OSF repository for this paper (see Data Availability statement in the main text).

For all of the following tables, effects are abbreviated as follows: ps - prestimulus amplitude; fq - unigram frequency; ss - speaker-based surprisal; sg – global surprisal; ep - epoch; cn - canonicity; id - ID; iaf – peak alpha frequency (IAF); slope – aperiodic (1/f) slope; ps2 - quadratic prestimulus amplitude; ss2 - quadratic speaker-based surprisal; ss3 - cubic speaker-based surprisal. Columns Est., SE, z and p provide the estimate, standard error, z-statistic and p-value for each of the fixed effects in the model. The σ columns include the variance components of the model, i.e. random intercepts and slopes by item, subject and channel.

Table S6: **Model summary for the best-fitting model including best-fitting model including 1/f slope in Experiment 2**

	Est.	SE	z	p	σ_{Item}	σ_{Subj}	σ_{Channel}
(Intercept)	-0.6441	0.0925	-6.96	$<$1e-11	0.9125	0.3089	0.1137
ps	4.1228	0.1668	24.71	$<$1e-99	0.4526	0.9505	0.2494
ss	-0.1721	0.0854	-2.02	0.0438	0.9965	0.2470	
cn	0.0990	0.0410	2.41	0.0159		0.1607	
fq x ss	-0.1735	0.0466	-3.73	0.0002			
ps x ep	0.1248	0.0237	5.28	$<$1e-06			
ss x ep	-0.1080	0.0538	-2.01	0.0447			
ps x fq x ep	-0.0511	0.0203	-2.52	0.0117			
fq x ss x cn	-0.1499	0.0385	-3.89	$<$1e-04			
fq x ss x sl	-0.0476	0.0205	-2.32	0.0201			
fq x ep x sl	0.0684	0.0199	3.43	0.0006			
ss x ep x sl	0.0738	0.0205	3.60	0.0003			
ps x cn x sl	0.0367	0.0154	2.39	0.0167			
ss x cn x sl	0.1440	0.0177	8.13	$<$1e-15			
ep x cn x sl	-0.1020	0.0179	-5.69	$<$1e-07			
ps x fq x ss x ep	0.0492	0.0201	2.45	0.0143			
ps x fq x ss x sl	-0.0581	0.0163	-3.56	0.0004			
ps x ss x ep x sl	-0.0484	0.0167	-2.89	0.0038			
fq x ss x ep x sl	0.0611	0.0211	2.90	0.0037			
ss x ep x cn x sl	-0.0657	0.0196	-3.36	0.0008			
ps x fq x ss x ep x cn	0.0446	0.0201	2.22	0.0264			
ps x fq x ep x cn x sl	0.1046	0.0166	6.31	$<$1e-09			
**ps x ss x ep x cn x sl**	-0.0421	0.0173	-2.44	0.0148			
**fq x ss x ep x cn x sl**	-0.0472	0.0210	-2.24	0.0248			
Residual	4.4098						

Table S7: **Model summary for the best-fitting model including best-fitting model including IAF in Experiment 2**

	Est.	SE	z	p	σ_{Item}	σ_{Subj}	σ_{Channel}
(Intercept)	-0.6491	0.0951	-6.82	<1e-11	0.9339	0.3293	0.1128
ps	4.1145	0.1647	24.99	<1e-99	0.4483	0.9330	0.2527
ss	-0.1771	0.0850	-2.08	0.0373	1.0015	0.2395	
cn	0.1207	0.0426	2.83	0.0046		0.1748	
fq x ss	-0.1447	0.0465	-3.11	0.0019			
ps x ep	0.1321	0.0237	5.58	<1e-07			
ss x ep	-0.1279	0.0538	-2.38	0.0175			
ps x fq x ep	-0.0608	0.0204	-2.98	0.0029			
fq x ss x cn	-0.1473	0.0384	-3.83	0.0001			
ss x ep x cn	-0.0761	0.0305	-2.50	0.0124			
fq x ss x iaf	-0.0714	0.0203	-3.51	0.0004			
ps x ep x iaf	0.0918	0.0157	5.86	<1e-08			
fq x ep x iaf	0.0975	0.0206	4.74	<1e-05			
ps x cn x iaf	0.0424	0.0149	2.85	0.0043			
fq x cn x iaf	0.0383	0.0193	1.99	0.0471			
ss x cn x iaf	0.0467	0.0183	2.55	0.0108			
ep x cn x iaf	-0.0365	0.0182	-2.00	0.0456			
ps x fq x ss x ep	0.0513	0.0202	2.55	0.0109			
ps x fq x ss x iaf	-0.0486	0.0164	-2.97	0.0030			
ps x fq x ep x iaf	-0.0423	0.0154	-2.74	0.0061			
ps x ss x ep x iaf	0.0377	0.0158	2.38	0.0172			
fq x ss x ep x iaf	0.1389	0.0185	7.52	<1e-13			
ps x fq x cn x iaf	-0.0455	0.0155	-2.93	0.0033			
ps x ss x cn x iaf	0.0320	0.0163	1.97	0.0492			
fq x ss x cn x iaf	-0.0466	0.0199	-2.34	0.0194			
ps x ep x cn x iaf	0.0807	0.0159	5.06	<1e-06			
fq x ep x cn x iaf	0.0487	0.0211	2.31	0.0211			
ss x ep x cn x iaf	-0.0432	0.0198	-2.18	0.0291			
ps x fq x ss x ep x cn	0.0453	0.0200	2.27	0.0234			
**ps x fq x ss x ep x iaf**	0.0415	0.0148	2.80	0.0052			
Residual	4.4058						

Table S8: **Model summary for the best-fitting model including best-fitting model including ID in Experiment 2**

	Est.	SE	z	p	σ_{Item}	σ_{Subj}	$\sigma_{Channel}$
(Intercept)	-0.6306	0.0983	-6.41	<1e-09	0.9838	0.3323	0.1138
ps	4.1320	0.1665	24.81	<1e-99	0.4553	0.9475	0.2518
cn	0.1092	0.0435	2.51	0.0120		0.1757	
fq x ss	-0.1724	0.0483	-3.57	0.0004			
ps x ep	0.1224	0.0235	5.20	<1e-06			
ps x fq x ep	-0.0538	0.0202	-2.66	0.0078			
fq x ss x cn	-0.1741	0.0396	-4.40	<1e-04			
ps x fq x id	-0.0432	0.0161	-2.68	0.0073			
ps x ss x id	-0.0424	0.0160	-2.65	0.0080			
ps x ep x id	0.0540	0.0173	3.12	0.0018			
fq x ep x id	-0.0478	0.0207	-2.31	0.0211			
ps x cn x id	0.0386	0.0157	2.46	0.0140			
fq x cn x id	-0.0673	0.0193	-3.49	0.0005			
ps x fq x ss x ep	0.0511	0.0200	2.56	0.0104			
ps x ss x ep x id	0.0358	0.0173	2.07	0.0388			
fq x ss x ep x id	0.0506	0.0212	2.39	0.0167			
ps x fq x cn x id	-0.0394	0.0162	-2.44	0.0147			
fq x ss x cn x id	-0.0743	0.0197	-3.78	0.0002			
ps x ep x cn x id	0.0545	0.0170	3.21	0.0013			
ss x ep x cn x id	-0.0748	0.0202	-3.70	0.0002			
ps x fq x ss x ep x cn	0.0502	0.0201	2.50	0.0124			
ps x fq x ss x cn x id	0.0392	0.0175	2.24	0.0248			
ps x fq x ep x cn x id	-0.0485	0.0174	-2.78	0.0054			
ps x ss x ep x cn x id	-0.0472	0.0173	-2.72	0.0065			
**ps x fq x ss x ep x cn x id**	-0.0534	0.0181	-2.95	0.0032			
Residual	4.4076						

3.3.1 Combined analysis: Experiments 1 and 2

Table S9: **Model summary for the best-fitting model including 1/f slope in the combined analysis of Experiments 1 and 2**

	Est.	SE	z	p	σ_{Item}	σ_{Subj}	σ_{Channel}
(Intercept)	-0.4944	0.0642	-7.70	$<$1e-13	0.6854	0.3366	0.1130
ps	3.5160	0.1200	29.31	$<$1e-99	0.4416	0.8940	0.2545
ss	-0.1766	0.0573	-3.08	0.0020	0.8111	0.2577	
ss2	-0.0343	0.0174	-1.97	0.0489			
ss3	0.0171	0.0084	2.04	0.0418			
ps x ep	0.1816	0.0176	10.34	$<$1e-24			
fq x ep	0.1829	0.0268	6.81	$<$1e-11			
ss x ep	-0.1009	0.0254	-3.97	$<$1e-04			
ps x cn	0.0282	0.0116	2.43	0.0151			
ss x cn	0.0518	0.0207	2.50	0.0125			
ep x cn	-0.1039	0.0196	-5.31	$<$1e-06			
ps x ss x ep	-0.0369	0.0134	-2.75	0.0060			
fq x ss x cn	0.0549	0.0256	2.15	0.0319			
fq x ep x cn	0.0683	0.0248	2.76	0.0059			
ss x ep x cn	-0.0546	0.0181	-3.02	0.0025			
ps x fq x sl	-0.0386	0.0114	-3.38	0.0007			
fq x ss x sl	-0.0452	0.0139	-3.26	0.0011			
ss x ep x sl	0.0881	0.0145	6.09	$<$1e-08			
ss x cn x sl	0.1031	0.0128	8.07	$<$1e-15			
ep x cn x sl	-0.0430	0.0143	-3.02	0.0026			
ps x fq x ss x ep	0.0571	0.0140	4.09	$<$1e-04			
ps x fq x ss x cn	0.0526	0.0134	3.93	$<$1e-04			
fq x ss x ep x cn	0.0955	0.0208	4.60	$<$1e-05			
ps x fq x ss x sl	-0.0343	0.0117	-2.94	0.0033			
ps x ss x ep x sl	-0.0387	0.0111	-3.50	0.0005			
fq x ss x cn x sl	-0.0268	0.0134	-2.00	0.0457			
ss x ep x cn x sl	-0.0598	0.0116	-5.16	$<$1e-06			
ps x fq x ss x ep x cn	0.0290	0.0134	2.16	0.0310			
**ps x fq x ss x ep x sl**	0.0352	0.0113	3.11	0.0019			
**ps x ss x ep x cn x sl**	-0.0613	0.0110	-5.58	$<$1e-07			
Residual	4.2786						

Table S10: **Model summary for the best-fitting model including IAF in the combined analysis of Experiments 1 and 2**

	Est.	SE	z	p	σ_{Item}	σ_{Subj}	$\sigma_{Channel}$
(Intercept)	-0.5033	0.0642	-7.84	<1e-14	0.6987	0.3332	0.1135
ps	3.5102	0.1213	28.93	<1e-99	0.4460	0.9068	0.2558
ss	-0.1711	0.0582	-2.94	0.0033	0.8410	0.2563	
ss2	-0.0401	0.0175	-2.29	0.0220			
ss3	0.0192	0.0084	2.27	0.0230			
ps x ep	0.1650	0.0177	9.32	<1e-19			
fq x ep	0.1879	0.0270	6.96	<1e-11			
ss x ep	-0.0798	0.0255	-3.12	0.0018			
ss x cn	0.0428	0.0207	2.07	0.0387			
ep x cn	-0.1098	0.0195	-5.63	<1e-07			
ss x paf	0.0829	0.0308	2.69	0.0071			
ps x fq x ep	-0.0337	0.0142	-2.36	0.0180			
fq x ss x cn	0.0614	0.0257	2.39	0.0169			
fq x ep x cn	0.0652	0.0249	2.62	0.0088			
ss x ep x cn	-0.0581	0.0180	-3.22	0.0013			
ps x fq x paf	-0.0365	0.0115	-3.16	0.0016			
fq x ss x paf	-0.0613	0.0136	-4.50	<1e-05			
ss x cn x paf	0.0400	0.0128	3.13	0.0018			
ps x fq x ss x ep	0.0477	0.0140	3.39	0.0007			
ps x fq x ss x cn	0.0514	0.0134	3.84	0.0001			
fq x ss x ep x cn	0.1017	0.0209	4.87	<1e-05			
ps x fq x ep x paf	-0.0249	0.0112	-2.23	0.0259			
fq x ss x ep x paf	0.0471	0.0122	3.86	0.0001			
ps x fq x cn x paf	-0.0339	0.0115	-2.95	0.0032			
ps x ss x cn x paf	0.0289	0.0113	2.55	0.0108			
fq x ss x cn x paf	-0.0539	0.0133	-4.05	<1e-04			
ps x fq x ss x ep x cn	0.0273	0.0135	2.03	0.0423			
**ps x fq x ss x ep x paf**	0.0424	0.0109	3.88	0.0001			
ps x fq x ep x cn x paf	0.0326	0.0112	2.91	0.0036			
**fq x ss x ep x cn x paf**	-0.0432	0.0118	-3.65	0.0003			
Residual	4.2795						

Table S11: **Model summary for the best-fitting model including ID in the combined analysis of Experiments 1 and 2**

	Est.	SE	z	p	σ_{Item}	σ_{Subj}	$\sigma_{Channel}$
(Intercept)	-0.5111	0.0663	-7.71	$<$1e-13	0.7140	0.3505	0.1133
ps	3.5227	0.1192	29.56	$<$1e-99	0.4447	0.8821	0.2547
ss	-0.1554	0.0601	-2.59	0.0097	0.8870	0.2581	
ss2	-0.0474	0.0177	-2.69	0.0072			
ss3	0.0209	0.0085	2.46	0.0139			
ps x ep	0.1733	0.0183	9.49	$<$1e-20			
fq x ep	0.1488	0.0274	5.42	$<$1e-07			
ss x ep	-0.0615	0.0259	-2.37	0.0179			
ep x cn	-0.0802	0.0201	-4.00	$<$1e-04			
ps x id	0.2279	0.0958	2.38	0.0174			
ps x fq x ss	0.0309	0.0139	2.22	0.0267			
ps x fq x ep	-0.0426	0.0146	-2.92	0.0035			
ps x fq x cn	0.0284	0.0143	1.99	0.0461			
fq x ss x cn	0.0660	0.0262	2.52	0.0117			
fq x ep x cn	0.0579	0.0252	2.30	0.0216			
ss x ep x cn	-0.0669	0.0183	-3.65	0.0003			
ps x fq x id	-0.0460	0.0121	-3.81	0.0001			
fq x ep x id	-0.0706	0.0152	-4.64	$<$1e-05			
ep x cn x id	0.0700	0.0145	4.82	$<$1e-05			
ps x fq x ss x ep	0.0518	0.0142	3.65	0.0003			
ps x fq x ss x cn	0.0519	0.0137	3.80	0.0001			
fq x ss x ep x cn	0.0773	0.0211	3.66	0.0002			
ps x fq x ep x id	-0.0359	0.0123	-2.93	0.0034			
fq x ss x ep x id	0.0410	0.0132	3.11	0.0018			
ps x fq x cn x id	-0.0292	0.0120	-2.43	0.0152			
ps x ss x cn x id	0.0378	0.0117	3.24	0.0012			
fq x ss x cn x id	-0.0384	0.0133	-2.88	0.0040			
ps x ep x cn x id	-0.0289	0.0121	-2.38	0.0173			
ps x fq x ss x ep x cn	0.0335	0.0139	2.42	0.0155			
ps x ss x ep x cn x id	0.0304	0.0114	2.66	0.0077			
fq x ss x ep x cn x id	0.0274	0.0125	2.19	0.0284			
**ps x fq x ss x ep x cn x id**	-0.0239	0.0120	-1.99	0.0470			
Residual	4.2796						

REFERENCES

Bornkessel, I. D., Fiebach, C. J., and Friederici, A. D. (2004). On the cost of syntactic ambiguity in human language comprehension: An individual differences approach. *Cognitive Brain Research* 21, 11–21

Gibson, E. (1998). Linguistic complexity: Locality of syntactic dependencies. *Cognition* 68, 1–76

Landauer, T. K., Foltz, P. W., and Laham, D. (1998). An introduction to latent semantic analysis. *Discourse Processes* 25, 259–284. doi:10.1080/01638539809545028

Wolff, S., Schlesewsky, M., Hirotani, M., and Bornkessel-Schlesewsky, I. (2008). The neural mechanisms of word order processing revisited: Electrophysiological evidence from Japanese. *Brain and Language* 107, 133–157. doi:10.1016/j.bandl.2008.06.003
